# Characterization of the heterogeneity of endothelial cells in bleomycin-induced lung fibrosis using single-cell RNA sequencing

**DOI:** 10.1007/s10456-021-09795-5

**Published:** 2021-05-24

**Authors:** Xiucheng Liu, Xichun Qin, Hao Qin, Caili Jia, Yanliang Yuan, Teng Sun, Bi Chen, Chang Chen, Hao Zhang

**Affiliations:** 1 Thoracic Surgery Laboratory, the First College of Clinical Medicine, Xuzhou Medical University, Xuzhou, 221006 Jiangsu China; 2 Department of Thoracic Surgery, Affifiliated Hospital of Xuzhou Medical University, 99 West Huaihai Road, Xuzhou, 221006 Jiangsu China; 3 Department of Thoracic Surgery, Shanghai Pulmonary Hospital, Tongji University School of Medicine, Shanghai, 200433 China; 4 Shanghai Engineering Research Center of Lung Transplantation, Shanghai, 200433 China; 5 Department of Respiratory and Critical Care Medicine, Affiliated Hospital of Xuzhou Medical University, Xuzhou, 221000 China

**Keywords:** Idiopathic pulmonary fibrosis, Endothelial cells, Extracellular matrix, Cell–cell interaction

## Abstract

The loss of normal alveolar capillary and deregulated angiogenesis occurs simultaneously in idiopathic pulmonary fibrosis (IPF); however the contributions of specific endothelial subpopulations in the development of pulmonary fibrosis are poorly understood. Herein, we perform single-cell RNA sequencing to characterize the heterogeneity of endothelial cells (ECs) in bleomycin (BLM)-induced lung fibrosis in rats. One subpopulation, characterized by the expression of Nos3 and Cav1, is mostly distributed in non-fibrotic lungs and also highly expresses genes related to the “response to mechanical stimulus” and “lung/heart morphogenesis” processes. Another subpopulation of ECs expanded in BLM-treated lungs, characterized by Cxcl12, is observed to be closely related to the pro-fibrotic process in the transcriptome data, such as “regulation of angiogenesis,” “collagen binding,” and “chemokine activity,” and spatially localized to BLM-induced neovascularization. Using CellPhoneDB software, we generated a complex cell–cell interaction network, which predicts the potential roles of EC subpopulations in recruiting monocytes, inducing the proliferation of fibroblasts and promoting the production and remolding of the extracellular matrix (ECM). Taken together, our data demonstrate the high degree of heterogeneity of ECs in fibrotic lung and it is proposed that the interaction between ECs, macrophages, and stromal cells contributes to pathologic IPF.

**Supplementary Information:**

The online version contains supplementary material available at 10.1007/s10456-021-09795-5.

## Introduction

Idiopathic pulmonary fibrosis (IPF), a common form of interstitial lung disease, is a chronic, debilitating, and progressive lung disease with a poor prognosis [1]. Epidemiological studies have revealed that the incidence and prevalence of IPF is increasing worldwide, and this may be a consequence of multiple interacting genetic and environmental risk factors [2]. Currently, there is no comprehensive understanding of IPF pathogenesis. Recent literature defines the loss of alveolar epithelial cells, the progressive deposition of the extracellular matrix (ECM), and deregulated angiogenesis as the core factors in the destruction of functional alveolar units [3–5]. However, previous conventional approaches have failed to detect and quantify the detailed contributions of altered cell types (epithelial cells, myofibroblasts, alveolar macrophages (AMs), endothelial cells (ECs)) in the IPF progression.

Single-cell RNA sequencing (scRNA-seq) has shown the potential to overcome the large-scale changes and spatial heterogeneity in cell types and allowed the reliable identification of related cell populations and confirmation of the complex molecular procedures in IPF. Xu and co-workers have profiled the roles of epithelial cells in IPF and identified an additional atypical transitional cell which contributes to the pathological processes [6]. ScRNA-seq has also demonstrated the diversity of stromal cells and their contributions to ECM expansion in human and mouse lung fibrosis [7–9]. Monocyte-derived macrophages transitioning to an alveolar form have also been localized to the fibrotic niche and exerted a pro-fibrotic effect [10]. Overall, these data provide detailed maps of single-cell transcriptomes in fibrotic lung and brought unprecedented insights into the central roles of epithelial cells, myofibroblasts, and macrophages in IPF pathogenesis. However, no studies to date have specifically focused on the role of ECs, which are intrinsically associated with fibrogenic progression in pulmonary fibrosis.

In normal lungs, contiguous capillaries occupy virtually all of the alveolar walls and are responsible for gas exchange. It has been recognized that the loss of pulmonary microvascular endothelial cells (PMVECs) and deregulated angiogenesis are the critical biological processes of pulmonary fibrosis [11–13]. Over the last decade, new data have shown proliferation of PMVECs with excessive formation of alveolar capillaries in the early pathological process of IPF, and angiogenesis inhibitor (nintedanib, kallistatin, endostatin) treatments can effectively ameliorate ECM accumulation and restore alveolar structure [14]. In other words, these revascularizations are often suboptimal due to an inability to reconstruct functional alveolar units that allow normal gas exchange. Overall, studies of ECs and their role in IPF appear to have lagged behind other cell studies perhaps due to the complexity of the lung with its two different circulatory beds.

To develop a more comprehensive understanding of the heterogeneity of ECs in IPF and explore the cross-talk between ECs, AMs, stromal cells, and their surrounding ECM, we performed scRNA-seq to identify ECs in mixed samples of uninjured and bleomycin (BLM)-treated rat lungs. The results indicated that increased cluster *a* ECs in BLM-treated lungs were enriched for processes associated with lung fibrosis, such as “chemotaxis activity,” “VEGF production,” “collagen binding,” and “ECM constituent.” In contrast, cluster *d* ECs were enriched in the control lungs and highly expressed genes related to “response to mechanical stimulus/cellular response to mechanical stimulus” and “lung/heart morphogenesis” programs. Notably, results of proximity ligation in situ hybridization showed that Nos3 + Cav1 + ECs were widely distributed in lung and heart tissue, but rarely in liver, brain, kidney, and fibrotic lung. It implies that the PMVECs adapt to continuous mechanical stimulation to acquire a unique phenotype and mediate unknown but important biological signals. In addition, in the context of pulmonary fibrosis, we further discuss the communication between ECs, macrophages, and stromal cells and emphasize the importance of ECs in recruiting monocytes, inducing the proliferation of fibroblasts and promoting the production and remolding of ECM.

## Methods

### Animal and treatment

Sprague Dawley (SD) rats (250 ± 20 g, at 8–10 weeks age) were obtained from the Experimental Animal Center of Xuzhou Medical College. The rats were kept on a 12-h light–dark cycle with free access to food and water. All experiments were performed in adherence with the National Institutes of Health (NIH Publication, 8th Edition, 2011) guidelines for the use of laboratory animals. The care and experimental protocols for rats were approved by the Animal Care and Use Committee of Xuzhou Medical University.

After adequate anesthesia (intraperitoneal injection of sodium pentobarbital; 60 mg/kg), BLM (Hanhui Pharmaceutical, China) was administered by oral tracheal intubation via a 16-gauge catheter. One dose of BLM (5 mg/kg, the BLM-treated group) or an equal volume of saline (the control group) was administered via the catheter. The rats were rotated immediately after instillation to ensure thorough drug distribution in the lungs. All rats were killed by cervical dislocation and lungs were harvested 28 days after intratracheal BLM or saline treatment.

### Measurement of lung fibrosis

Lung fibrosis was determined by Masson’s trichrome (MTC) staining (Solarbio Life Sciences, cat# G1345). The fibrotic area was determined by calculating the area of positive MTC staining region (blue).

### Single cell suspension

Rat lungs were harvested after perfusion through the right ventricle with PBS and 3 mm^3^ lung tissues were taken for digestion. Single-cell suspensions were generated as previously reported [15]. Briefly, minced tissues were placed in a digestion solution containing 480 U/ml Collagenase Type I (Sigma, cat #C0130), 50 U/ml Dispase (Collaborative Biosciences), and 0.33 U/ml DNase (Roche). It was allowed to incubate in a 37 ℃ water bath with frequent agitation for 45 min. Cell solution was filtered through 70 μm cell strainer (BD Biosciences). Red blood cell lysis solution (Thermo Fisher, cat #00–4333-57) was used to remove blood cells. Countess ® II Automated Cell Counting Chamber slides (Thermo Fisher, USA) were used to count living cells.

### ScRNA-seq library preparation and sequencing

Single cell capture was achieved by random distribution of a single cell suspension across approximately 200,000 microwells. Beads with unique molecular identifier (UMI) and cell barcodes were loaded close to saturation, so that each cell was paired with a bead in a microwell. After incubation with cell lysis buffer, polyadenylated RNA molecules were hybridized to the beads. Beads were retrieved into a single tube for reverse transcription. During synthesis, each cDNA molecule was tagged on the 5’ end (i.e., the 3’ end of a messenger RNA transcript) with UMI and cell label indicating its cell of origin. Whole transcriptome libraries were prepared using the BD Rhapsody system (BD Biosciences) as previously reported [16, 17]. Briefly, Rhapsody beads were used for second-strand cDNA synthesis, adaptor ligation, and universal amplification. Sequencing libraries were prepared using random priming PCR of the whole transcriptome amplification products to enrich the 3’end of the transcripts linked with the cell label and UMI. Sequencing libraries were quantified using a high sensitivity DNA chip on a Bioanalyzer 2100 and the qubit high sensitivity DNA assay. The libraries were sequenced on NovaSeq6000 (Illumina) using 2 × 150 chemistry.

### Cell type annotation of single cells

SingleR and known marker genes were used to annotate each single cell independently as previously reported [18]. Firstly, we calculated a spearman coefficient for single cell and then performed correlation analysis on highly variable genes among cell types in the reference data set. Multiple correlation coefficients were made per cell type according to the named annotations of the reference data set and aggregated to provide a single value per cell type per single cell. Secondly, the least relevant cell types (or values more than 0.05 below the top value) were successively removed, and this action was repeated until annotation of the target cells was achieved.

### Identification of marker genes

The Wilcoxon rank sum test was performed to determine genes differentially expressed between clusters as described previously [19]. Differentially expressed genes were scored by group one vs. the remainder. Genes that were specifically and highly expressed (logFC > 0.25) and expressed in at least 20% of cells were considered to be ideal marker genes for each cluster.

### CellPhoneDB annotation of cell–cell communication

CellPhoneDB software was used to systematically analyze cell–cell communication molecules. Lists of interacting protein chains/receptor-ligands complexes are available at https://www.cellphonedb.org/downloads. Pairwise comparisons between all cell types were conducted in this study. Only receptors and ligands expressed in more than 10% of the cells in the specific cluster were considered to indicate relevant interactions between cell types. Interactions that were highly enriched between cell populations based on the number of significant pairs were emphasized and those which were biologically relevant were selected manually.

### Immunohistochemical staining (IHC)

After antigen retrieval using EDTA, the specimens were blocked with goat serum for 20 min before applying the primary antibody. Specimens were incubated with anti-CD31 (Servicebio, cat# GB13428) for 12 h at 4 °C. Next, the sections were washed twice with phosphate buffer saline (PBS) and subsequently incubated with HRP polymer-conjugated secondary antibody at room temperature for 30 min. Finally, the microarrays were stained with 3, 3-diaminobenzidine solution and hematoxylin. The slides were photographed with an inverted microscope (Olympus).

### Fluorescence probe-FISH and Immunofluorescence

Sections were covered with proteinase K (20 μg/ml) working solution at 37 ℃ for 60 min. After washing with PBS (5 min × 3), pre-hybridization solution was added to the sections and incubated at 37 ℃ for 1 h. Sections were further incubated with probe hybridization solution (Nos3, Cav1, Cxcl12, Mki67) in a humidity chamber overnight. Before applying the primary antibody, sections were blocked with serum at room temperature for 30 min. PBS solution containing anti-VE-cadherin (Abcam, cat# ab33168) was added and incubated at 4 °C overnight. Samples were then washed with PBS at room temperature (5 min × 3). After washing, sections were incubated for 50 min with secondary antibody at room temperature. Nuclei were stained with DAPI (4’, 6-diamidino-2-phenylindole, Key-Gen Biotech, catalog #KGA215–10). Then, the sections were observed under a fluorescence microscope (Olympus). Fluorescence was calculated by viewing five randomly selected fields for each group. Image-Pro Plus software (v6.0, Media Cybernetics, Inc) was used for quantification.

### Statistical analysis

The statistical methods used for each analysis are described in the above “Methods” sections and in the figure legends.

## Results

### Single-cell atlas of BLM-treated and control rat lungs

**Fig. 1:**
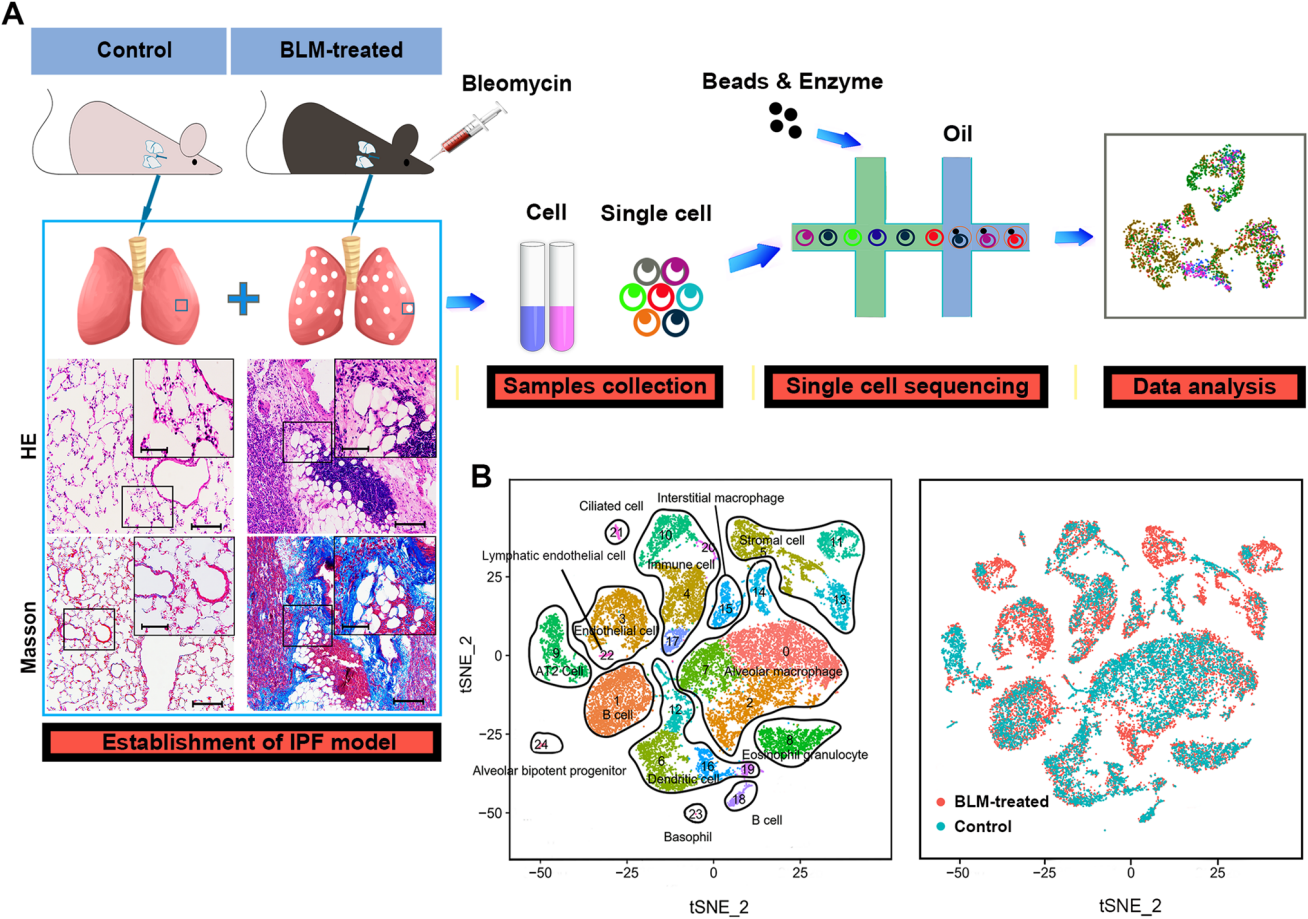
Single-cell atlas of BLM-treated and control rat lungs. **a** Schematic of workflow for scRNA-seq using the BD Rhapsody system. Representative H&E and MTC staining of rat lungs, bar (up) = 50 μm, bar (down) = 100 μm. **b** T-distributed stochastic neighborhood embedding (t-SNE) plot of 25,547 cells to visualize cell-type clusters based on the expression of known marker genes. **c** Injury status

Appreciable amounts of lung fibrosis and angiogenesis were observed at day 28 in rats treated with BLM (Supplementary Fig. 1). Single-cell suspensions were generated from three BLM-treated (day 28) and three vehicle control rat lungs. ScRNA-seq was performed on the samples using the BD Rhapsody system (Fig. 1a). In total, 25,547 single-cell transcriptomes were profiled (10,538 cells from the control lungs, 15,009 cells from the BLM-treated lungs). Using previous canonical markers, we defined 25 clusters of cells and visualized these cells in two dimensions by t-distributed stochastic neighborhood embedding (t-SNE), as indicated (Fig. 1b, c). Overall, each cluster was identified both in the control and BLM-treated lungs, but in different proportion, especially ECs, stromal cells, and macrophages. In comparison with the control lungs, the populations of ECs (cluster 3, 22; 11.67% versus 2.17%), stromal cells (cluster 5, 11, 13; 17.29% versus 4.54%), and ciliated cells (0.73% versus 0.08%) were increased. The proportion of macrophage cells (cluster 0, 2, 7, 14, 15) decreased from 40.34% [BLM-treated] to 27.50% [control] (Supplementary Fig. 2). The top three differentially expressed genes of these clusters are shown in Supplementary Fig. 3.

### The transcriptomic heterogeneity of ECs

**Fig. 2:**
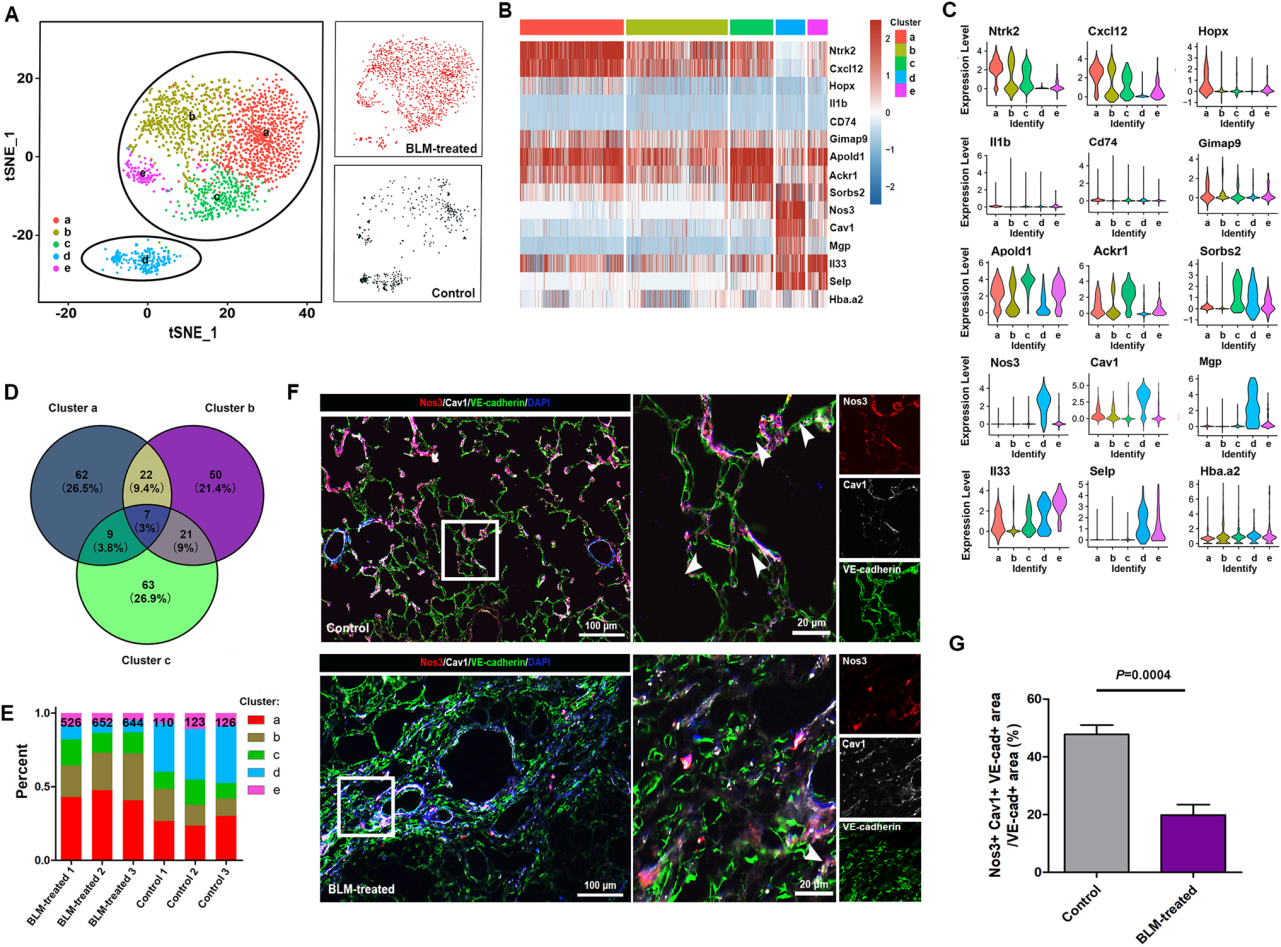
The transcriptomic heterogeneity of ECs. **a** Clustering 2,181 ECs (left) and annotation by injury condition (right). **b** Heatmap: ECs cluster marker genes (top 3, color coded by cluster and condition), exemplar genes labeled (right), cells columns, genes rows. **c** The expression of the top three differentially expressed genes within five recognized EC clusters. **d** Venn diagram of the top 100 differentially expressed genes in cluster *a*, *b*, and *c* ECs. **e** Fractions of ECs subpopulations in three control and three BLM-treated lungs. **f** Representative immunofluorescence image: Nos3 (red), Cav1 (white), VE-cadherin (green), DAPI (blue), bar (left) = 100 μm, bar (right) = 20 μm, the white arrows indicate Nos3 + Cav1 + ECs. **g** Quantitative analysis of Nos3 + Cav1 + ECs, Data is shown as Mean ± SEM. Statistical analysis using Mann–Whitney two-tailed test (n = 5)

To further determine the detailed role of ECs (cluster *3*) in fibrotic lungs, we performed focused analyses on 2,181 ECs (479 cells from the control lungs, 1,702 cells from the BLM-treated lungs) and identified five distinct subtypes/subpopulations (Fig. 2a, Supplementary Fig. 4). Previously known marker genes for ECs, including CD31, CD34, CD144, Vegfr, Vwf, and Pecam1, failed to distinguish these subtypes. Here, subpopulation markers were identified and the top three differentially expressed genes are listed in Fig. 2b, c. The venn diagram shows that cluster *a*, *b*, and *c* ECs were distinct in the transcriptome, although there was a spatially close relation in t-SNE (Fig. 2d). Compared to samples from the control lungs, the number and proportions of clusters *a, b*, and* c* ECs were increased, while cluster *d* ECs decreased in the BLM-treated lungs (Fig. 2e).

Cluster *a* ECs expressed high levels of Ntkr2 and Cxcl12 and relatively low levels of Nos3, Cav1, Mgp, and Selp. Compared with other subpopulations, cluster *b* ECs lacked markedly different marker genes. Cluster *c* expressed high levels of Ackr1, Apold1, and Vcam1 (Supplementary Fig. 5). Cluster *d* expressed high levels of Nos3, Cav1, and Mgp and relatively low levels of Ntkr2, Cxcl12, and Hopx. Cluster *e* expressed high levels of Il33 (encoding interleukin-33) and Selp, which were closely related to the interaction of activated ECs or platelets with leukocytes in the initial steps of inflammation.

Furthermore, the latest data shows that endothelial-mesenchymal transition (EndMT) is involved in the pathogenesis of fibrosis [20, 21]. The expression levels of EndMT-related genes were analyzed, and results indicated that there were no differences in the expressions of Vim, Cadh1, Cadh2, and Cdh5 among five EC subpopulations (Supplementary Fig. 6).

The highly sensitive FISH technique was used to determine whether scRNA-seq accurately localized specific gene expression to specific cell populations. RNA-ISH for Cxcl12 better identified cluster *a* ECs than Ntrk1 (neurotrophic receptor tyrosine kinase 2) since Ntrk1 also stained ECM (data not shown). Next, multiplexed immunofluorescence staining of Cxcl12-VE-cadherin and Nos3-Cav1-VE-cadherin was performed to examine the spatial localizations of cluster *a* and *e* ECs (Fig. 2f, Supplementary Fig. 7). In detail, Nos3 + Cav1 + ECs were topographically located in alveolar capillaries in the control lungs. Quantitative analysis showed that the number of Nos3 + Cav1 + ECs was significantly decreased in the fibrotic area (Fig. 2g). By contrast, BLM-treated lungs contained more Cxcl12 + ECs.

### The potential functional heterogeneity of ECs

**Fig. 3:**
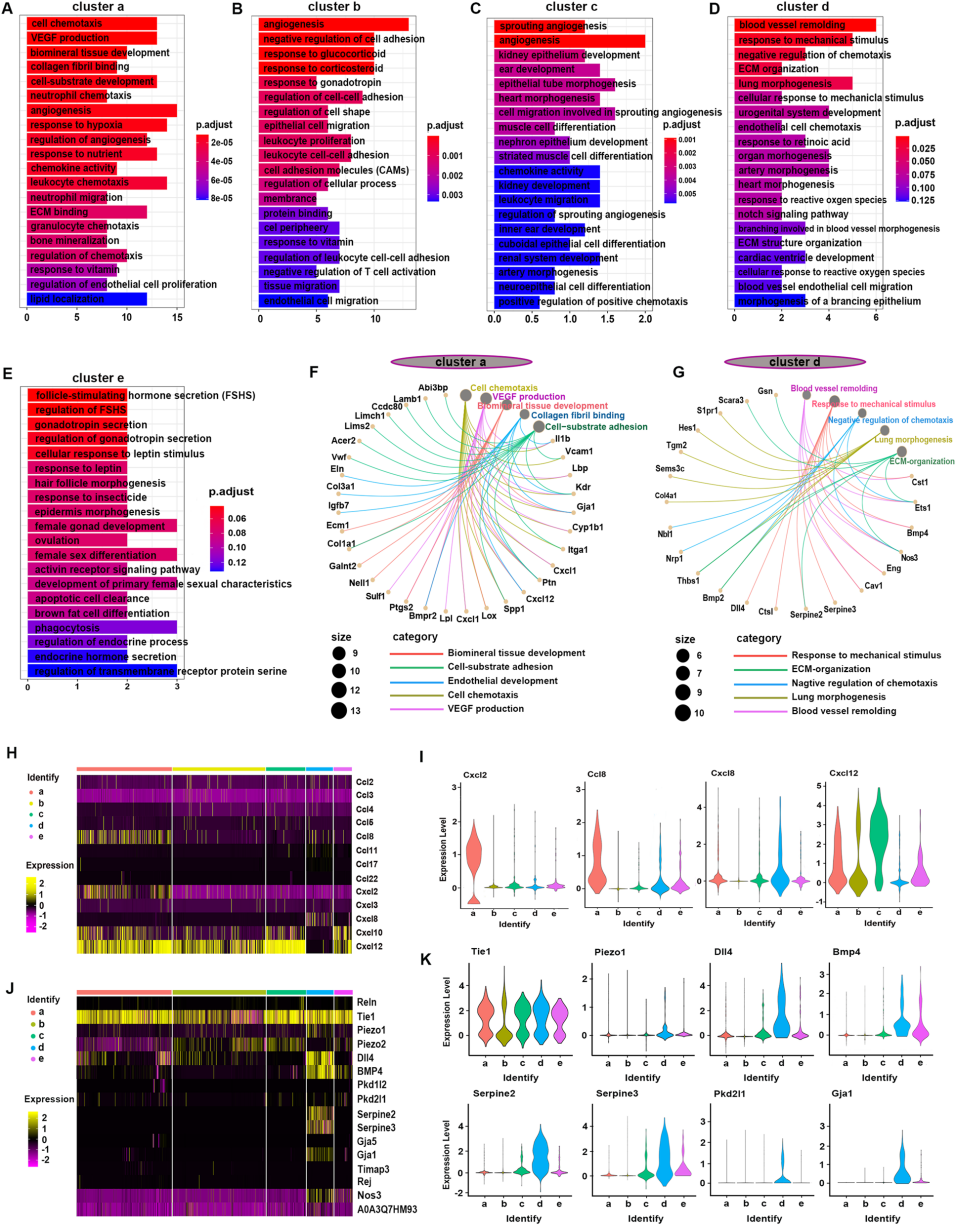
The potential functional heterogeneity of ECs. **a–e** The top 20 GO enrichment of cluster *a*–*e* ECs. **f** The top 5 Go enrichment network of cluster *a* ECs. **g** The top 5 Go enrichment network of cluster *d* ECs. **h** Heatmap depicting relative expression of known genes related to “chemotaxis activity”. **i** Violin plot showing the Cxcl2, Ccl8, Cxcl8, and Cxcl12 in EC subpopulations. **j** Heatmap depicting relative expression of known genes related to “response to mechanical stimulus” previously shown to be increased in fibrotic lung. **k** Violin plot showing the Tie1, Piezo1, Dll4, Bmp4, Serpine2, Serpine3, Pkd2l1, and Gja1 in EC subpopulations

We further performed Gene Ontology (GO) and Kyoto Encyclopedia of Genes and Genomes (KEGG) enrichment analyses on marker genes (top 100) of these EC clusters. Cluster *a* ECs were enriched for “cell chemotaxis,” “VEGF production,” “regulation of angiogenesis,” and “ECM binding” processes, and VEGF, Toll-like receptor, and TGF-beta signaling pathways (Fig. 3a, f, Supplementary Fig. 8A). Heatmap and violin plots for the expression levels of known chemotaxis activity-related genes showed that Ccl8, Cxcl2, Cxcl10, and Cxcl12 were highly expressed in cluster *a* ECs (Fig. 3h, i). In addition, cluster *a* ECs also express high levels of ECM-related genes*,* such as Eln, Col3a1, Igfbp7, and Lpl, indicating potential cross-talk between cluster *a* ECs and ECM-producing cells (Supplementary Fig. 9).

Enrichment analyses of highly expressed genes indicated increased “regulation of cell adhesion,” “response to corticosteroid,” and “epithelial cell migration” processes, and increased ErbB signaling pathway in cluster *b* ECs (Fig. 3b, Supplementary Fig. 8B). Cluster *c* ECs were enriched for “sprouting angiogenesis” and “epithelial tube morphogenesis” processes and pathways in cancer (Fig. 3c, Supplementary Fig. 8C). Cluster *e* ECs were enriched for “follicle-stimulating hormone secretion (FSHS),” “gonadotropin secretion,” and “cellular response to leptin stimulus” processes (Fig. 3e, Supplementary Fig. 8E).

Notably, cluster *d* ECs were enriched for “response to mechanical stimulus/cellular response to mechanical stimulus,” “lung/heart morphogenesis,” “blood vessel remolding,” and “cardiac ventricle development” processes (Fig. 3d, g, and Supplementary Fig. 8D). Known mechanical stimulation related genes, such as Cav1, Serpine2, Serpine3, Piezo1, Dll4, Tie1, BMP4, and Clu, were highly expressed in cluster *d* ECs (Fig. 3j, k). In addition, the plasma membrane proteins/receptors (e.g., Itga1, Icam1, Edn1, Aqp1, Tubb5, and Jam2) which were reported to play roles in transmitting mechanical stress into biochemical signaling cascades were observed to be highly expressed in cluster *d* ECs (Supplementary Fig. 10) [22–25]. Immunofluorescence staining showed that Nos3 + Cav1 + ECs could be identified in the lung and heart instead of kidney, brain, and liver (Supplementary Fig. 11).

### Single-cell profiling of macrophages and stromal cells

Previous genetic studies in rodents have revealed macrophage and stromal cell subpopulations orchestrating both fibrotic diseases progression and regression. In this study, five macrophage clusters and five stromal cell clusters were identified.

**Fig. 4:**
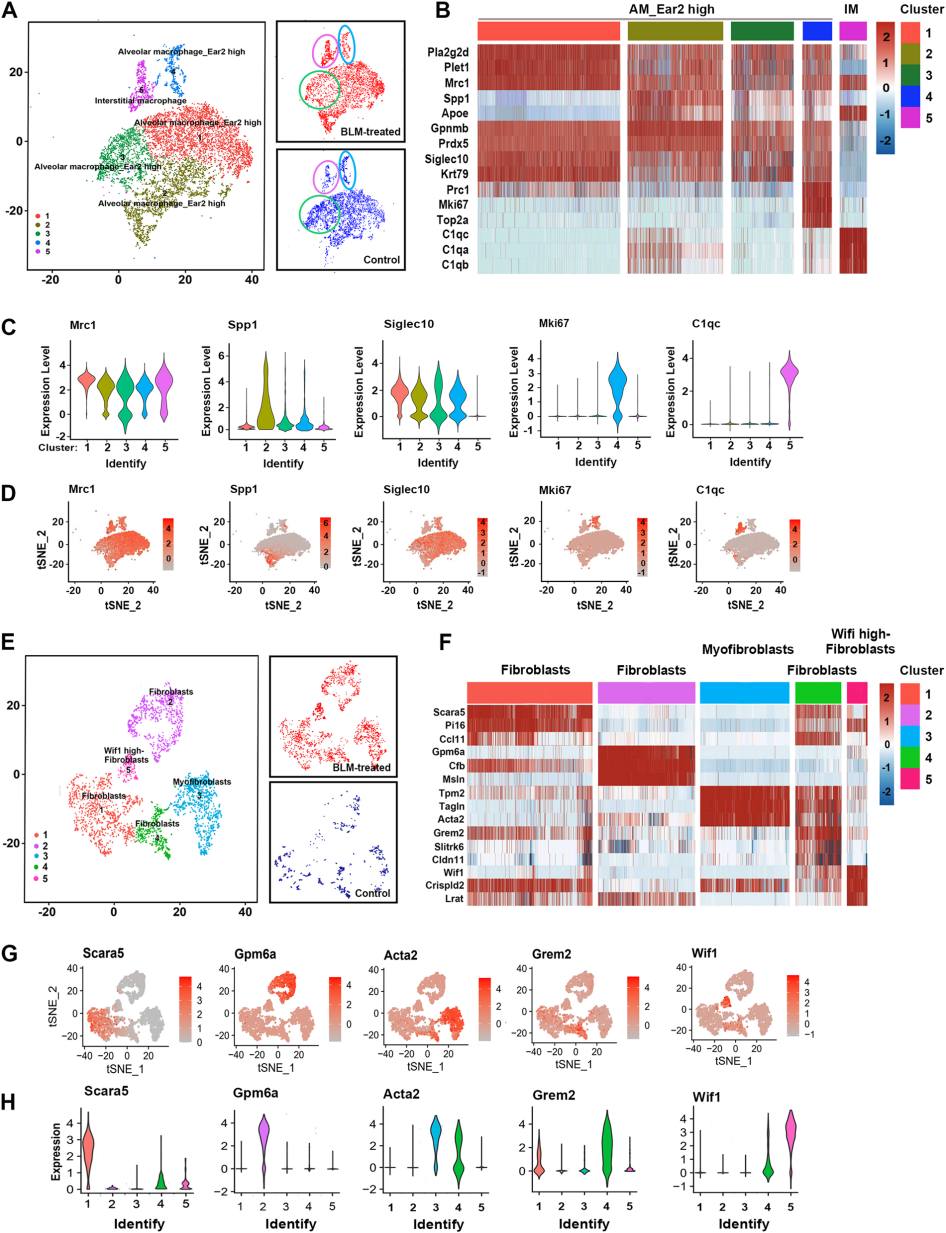
Single-cell profiling of macrophages and stromal cells. **a** Clustering and annotating 8,379 macrophages (left), annotating injury condition (right). **b** Heatmap: macrophage cluster marker genes (top 3, color coded by cluster and condition), exemplar genes labeled (right), cells are shown in columns, genes are shown in rows. **c**, **d** The expressions of selected marker genes across 5 macrophage subpopulations. **e** Re-clustering and annotating 3,067 stromal cells (left), annotating injury condition (right). **f** Heatmap: stromal cell cluster marker genes (top 3, color coded by cluster and condition), exemplar genes labeled (right), cells columns, genes rows. (G, H) The expressions of selected marker genes across 5 stromal cell subpopulations

Macrophages were annotated as Ear2 high-AMs (cluster *1*, *2*, *3*, *4*) and interstitial macrophages (IMs) (cluster *5*) (Fig. 4a–d). Cluster *1* macrophages expressed relatively high levels of Mrc1, as well as CD14, IL1B. GO analysis demonstrated that cluster *1* AMs was enriched for “chemokine receptor binding,” “monocyte chemotaxis,” and “complement activation” processes. Cluster *2* AMs highly expressed genes related to “inflammatory response,” “phagocytosis,” and “collagen metabolic processes,” such as Spp1, Trem2, Ctsl, Arg1, and Mmp14, suggesting that cluster *2* AMs may correspond to the scar-associated macrophage (SAMΦ) reported by Ramachandran et al. [10]. Cluster *3* AMs were enriched for processes relevant to responses to “glucocorticoid,” “collagen metabolic processes,” and “regulations of SMC proliferation.” Cluster *4* AMs highly expressed genes closely related to “mitosis” and “cell proliferation,” such as Mki67, Ckap2, and Smc4. The cluster *5* IMs were enriched for processes of “inflammatory response,” “chemokine activity,” and “the external side of the plasma membrane” (Supplementary Figs. 12–15).

**Fig. 5:**
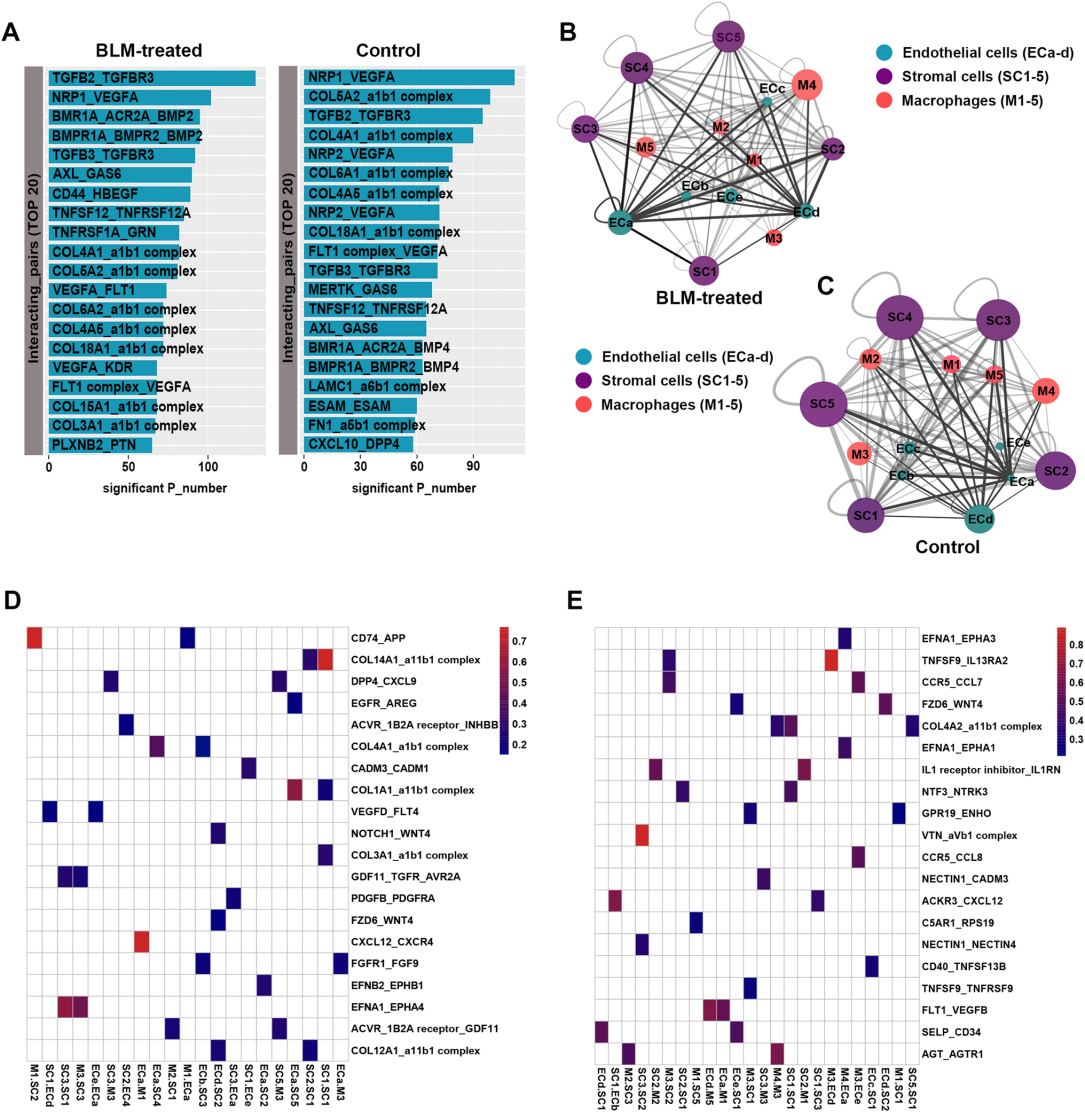
Cell communication predicted by CellPhoneDB. **a** The top 20 ligand-receptor pairs across ECs, macrophages, and stromal cells. **b** Network visualizing potential specific interactions in the BLM-treated lungs, in which nodes are selected clusters and edges represent the number of significant ligand–receptor pairs. The network layout was set to force-directed layout. **c** Network visualizing potential specific interactions in the control lungs. **d** Heatmap: Top 20 (p-value) ligand–receptor pairs. **e** Heatmap: Top 20 (p-value) ligand–receptor pairs

Re-clustering the 3,067 stromal cells revealed five discrete populations of fibroblasts (Fig. 5e–h). Cluster *1* expressed high levels of Scara5, Pi16 and the highest levels of Col1a1, Col3a1, and Cthrc1, suggesting that cluster *1* stromal cells may correspond to the collagen-producing Cthrc1 + cells as reported by Tsukui et al. [26]. Cluster *2* stromal cells expressed high levels of Gpm6a, in addition to lipofibroblast markers (e.g., Fabp4, Fabp5, and Krt79). Cluster *3* stromal cells were annotated as myofibroblasts given their high expression of Acta2, Actg2, and Myh11. Cluster *4* stromal cells expressed high levels of Grem2 and Coldn11. Cluster *5* stromal cells expressed high levels of genes related to angiogenesis (e.g., Vegfa, Reck, and Mmp2) (Supplementary Fig. 16). A heatmap of known ECM components and GO enrichment analysis of the highly expressed genes showed that all subtypes were enriched for ECM-related processes (Supplementary Fig. 17A, 17B). In addition, cluster *1*, *2*, and *5* stromal cells were enriched for processes of “response to VEGF stimulus” and “blood vessel remolding,” and cluster *2*, *4*, and *5* were closely relevant to “monocyte chemotaxis” (Supplementary Fig. 17C–17G). These data imply potential communication between stromal cells, ECs, and macrophages in fibrotic processes.

In addition, we found that SingleR failed to identify perivascular cells (PCs) and vascular smooth muscle cells (VSMCs) which are highly involved in both fibrosis and angiogenesis. Feature plots of known marker genes of VSMCs (Cnn1, Acta2, Tagln, Rgs5) and PCs (Cspg4, Trpc6, Pdgfrb) suggested that these cells may be included in stromal population (Supplementary Fig. 18).

### Cell communication predicted by CellPhoneDB

In order to systematically examine the interaction between ECs, macrophages, and stromal cells, the CellPhoneDB database of ligand–receptor interacting pairs developed by Roser et al. [27] was used in this study. Briefly, by identifying the expression levels of biologically relevant ligand–receptor complexes within cell populations of interest and using empirical shuffling to calculate which ligand–receptor pairs display significant cell-type specificity, we generated cell–cell communication networks in the control and the BLM-treated lungs. The common ligand–receptor pairs in both control and BLM-treated lungs were TGFB3_TGFBR3, NRP1_VEGFA, AXL_GAS6, and COL4A5_a1b1 complexes. In BLM-treated lungs, the increased ligand–receptor pairs were CD44_HBEGF, VEGFA_FLT1, and VEGFA_KDR (Fig. 5a). Interaction network analysis indicated potential cross-talk between ECs, macrophages, and stromal cells (Fig. 5b, c). The enrichment of CXCL12_CXCR4 (ECa_M1), COL1A1_a11b1 (ECa_F5), COL4A1_a1b1 complex (ECa_F4), FGFR1_FGF9 (ECa_F3), and VEGFD_FLT4 (ECe_ECa) in BLM-treated lungs further proved the potential role of cluster *a* ECs in recruiting circulating monocytes, inducing the proliferation of fibroblasts, and promoting the production of ECM (Fig. 5d). The enrichment of TNFSF9_IL13RA2 (M3_ECd), FZD6_WNT4 (ECd_F2), SELP_CD34 (ECd_F1), and FLT1_VEGFB (ECa_M5) suggested that cluster *d* ECs were closely involved in the biological process in the normal lungs (Fig. 5e).

## Discussion

The lung is a highly vascular organ responsible for efficient gas exchange. Understanding the role of ECs in the pathogenesis of fibrosis is important and current knowledge is limited. Here, our scRNA-seq data provide insight into the heterogeneity and plasticity of ECs in normal and fibrotic lungs and suggest that the potential cross-talk between ECs, macrophages, and stromal cells contributes to pathologic IPF.

A recent study reported that ECs from different tissues exhibited prominent transcriptomic heterogeneity to meet the distinct physiological needs [28]. Using scRNA-seq, Sébastien et al*.* [29] identified four capillary endothelial cell phenotypes in mice kidney, highlighting extensive heterogeneity of ECs within the cortex, glomeruli, and medulla. Similarly, Paul and colleagues identified four distinct EC subgroups from a living patient with IPF, but did not elaborate on their respective functions, phenotypes, or potential links with IPF progression [10].

In this study, we performed focused analyses on ECs and identified five distinct EC subpopulations. Cluster *a* ECs markedly expressed high levels of Cxcl12 and were expanded in the BLM-treated lung tissue. Further analysis showed that these cells were enriched for biological processes relevant to lung injury and fibrosis, such as “VEGF production,” “positive regulation of angiogenesis,” “monocytes chemotaxis,” and “ECM binding.” Thus, cluster *a* ECs exhibit a pro-fibrotic phenotype and may play a critical role in lung fibrosis through potential cross-linking with AMs and stromal cells. Cluster *c* ECs express high levels of Ackr1 and Vcam1 and may correspond to “large artery/vein” ECs as classified by Joanna et al. [30]. Cluster *e* ECs express high levels of Il33 and Selp and were enriched for “follicle-stimulating hormone secretion (FSHS)” process. FSH is a pituitary glycoprotein that regulates follicle maturation through its binding to follicle-stimulating hormone receptor (FSHR). Endothelial cells express FSHR, but its exact role in ECs remains unclear [31]. Recent data indicated that FSH stimulation promoted human umbilical vein endothelial cells migration but not proliferation [32], but the effect of FSH on pulmonary circulation needs to be further explored.

Cluster *d* ECs are mainly derived from the control lungs. These cells are not enriched for biological processes relevant to lung fibrosis, but markedly express high levels of genes that are closely associated with “responses to mechanical stimulus” and “lung/heart morphogenesis.” Current literature generally concurs that ECs can sense and translate mechanical signals into intracellular biochemical signals and mediate regulation of many physiological functions (e.g., cell adhesion, migration, proliferation, and wound healing process) [22, 33]. Studies by Chachisvilis et al*.* [34] also provided clear evidence that mechanotransduction in ECs can modulate endothelial permeability and is associated with the progression of atherosclerosis. The pulmonary circulation is constantly exposed to mechanical microenvironment. Therefore, we proposed that cluster *d* ECs may be the product of the mechanical microenvironment of the lungs and may contribute to the unique structure and support specific lung processes. This assumption is further supported by the following data: Nos3 + Cav1 + ECs can be found in the rat heart rather than the kidney or brain, which may be due to the fact that the heart is an organ that constantly generates mechanical signals to internal cells like the lung. The loss of cluster *d* ECs in the BLM-treated lungs is likely to be an important reason for the failure of revascularization of alveolar units.

ECs, macrophages, and stromal cells have close spatial connections and multi-directional intercellular communication in IPF lungs [35, 36]. In this study, five clusters of macrophages and five clusters of stromal cells were identified in fibrotic lungs. Cluster *2* AMs highly expressed genes related to wound healing and ECM deposition. In terms of transcriptional phenotype and potential functions, these cells correspond to a novel pro-fibrogenic scar-associated macrophage subpopulation in liver fibrosis [10]. Consistent with previous reports [37, 38], the data herein showed that fibroblasts and myofibroblasts may be the major collagen-producing cells. The transcriptomes of the five stromal cell clusters suggested that they were closely related to the production, decomposition, binding, and deposition of ECM. Moreover, the cluster *2* and *5* stromal cell showed high expression of genes relevant to “monocyte chemotaxis” and “response to VEGF stimulus” processes. These data further emphasize the important role of the cross-talk between ECs, macrophages, and stromal cells in regulating the progress of IPF.

To further elucidate the potential cross-talk that contributes to pathologic IPF, we performed CellphoneBD analysis and identified a wide range of classical receptor–ligand complexes within ECs, macrophages, and stromal cells subpopulations in both control and BLM-treated lungs. In BLM-treated lungs, the enrichment of CXCL12_CXCR4, CD74_APP, VEGFD_FLT4, FGFR1_FGF9, COL1A1_a11b1 complex, and COL4A1_a1b1 complex highlighted the “monocyte chemotaxis,” “regulation of proliferation and differentiation of fibroblasts,” and “ECM binding” activities of cluster *a* ECs. In addition, the enrichment of TNFSF9_IL13RA2, FLT1_VEGFB, and SELP_CD34 suggested that cluster *d* ECs were closely related to “innate immune response,” “cell–cell adhesion,” and “endothelium development.” Therefore, the important role of cluster *d* ECs in non-fibrosis lung should be considered.

Despite the significance of the findings in this study, there are several limitations that warrant discussion. Firstly, although some evidence supports the hypothesis that the special mechanical microenvironment leads to a unique ECs subpopulation, further studies are needed to characterize the evolutionary characteristics of this phenotype and to determine their involvement in the progression of IPF and related mechanisms in vivo and in vitro. Secondly, the genetic, anatomical, and behavioral differences between rodents and humans complicate the translation of results from mice to humans. Therefore, increased analysis based on samples from PF patients is necessary.

In conclusion, using scRNA-seq data and spatial mapping, the large degree of heterogeneity of ECs was identified and dissected into a multi-lineage interaction in BLM-treated and control lungs. These data provided important insights into the lineage relationships, homeostatic, and pathologic roles of ECs.

## Supplementary Information

Below is the link to the electronic supplementary material.

Supplementary file1 (DOCX 2906 kb)

## Data Availability

The authors declare that all data supporting the findings of this study are available in the paper and its Supplementary Information.
