# Supplementary material for: Characterization of the heterogeneity of endothelial cells in bleomycin-induced lung fibrosis using single-cell RNA sequencing
Source: Angiogenesis. 2021 May 24;24(4):809–21. doi: 10.1007/s10456-021-09795-5 (PMC8487874; doi:10.1007/s10456-021-09795-5)
Supplement: Supplementary file 1 — Supplementary file1 (DOCX 2906 kb) [file 10456_2021_9795_MOESM1_ESM.docx]

**Supplementary Figures**


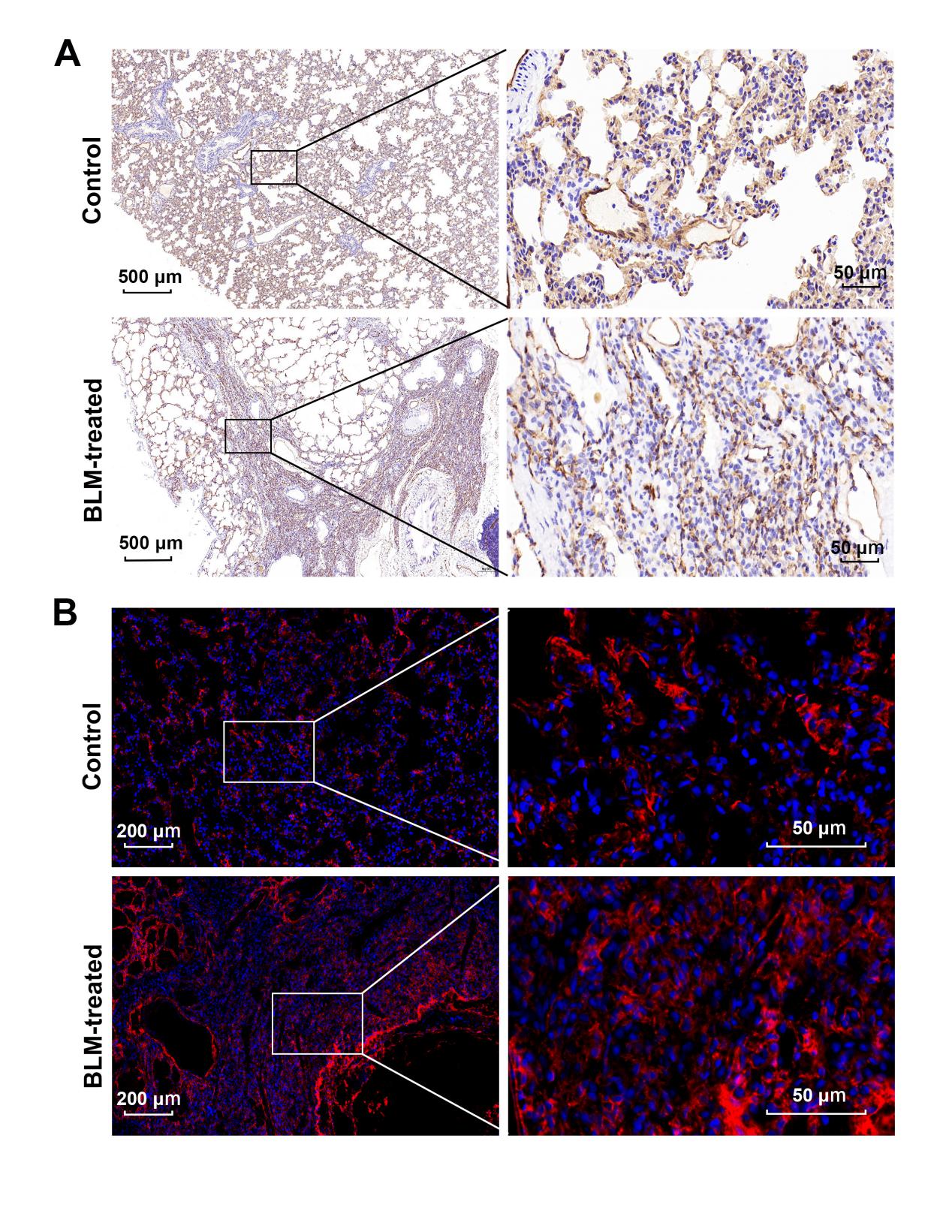


**Supplementary Figure 1. The deregulated angiogenesis in BLM-treated lungs.**

1. CD31 [immumohistochemical](C:/Program%20Files%20(x86)/Youdao/Dict/8.9.6.0/resultui/html/index.html#/javascript:;) [staining](C:/Program%20Files%20(x86)/Youdao/Dict/8.9.6.0/resultui/html/index.html#/javascript:;) in sections from lungs treated with saline and BLM (4 weeks), bar (left) = 500 μm, bar (right)=50 μm. (B) Immunofluorescence images of CD31 of lung sections. red (CD31), blue (DAPI), bar(left)= 200 μm, bar (right)=50 μm.


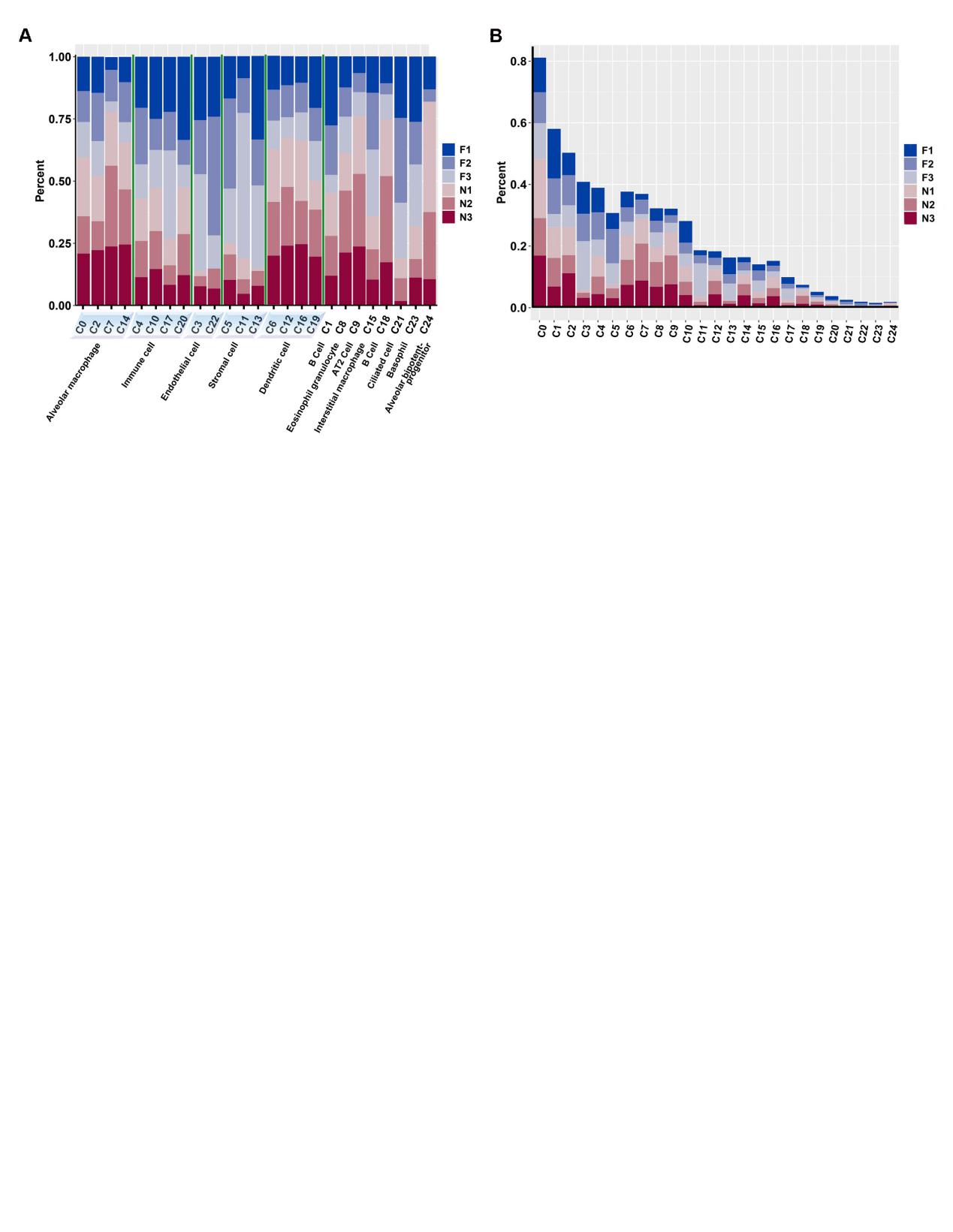


**Supplementary Figure 2. Cell type percent statistics.**

(A, B) The proportion of cells that contributed to each cluster by each sample, colored by samples.


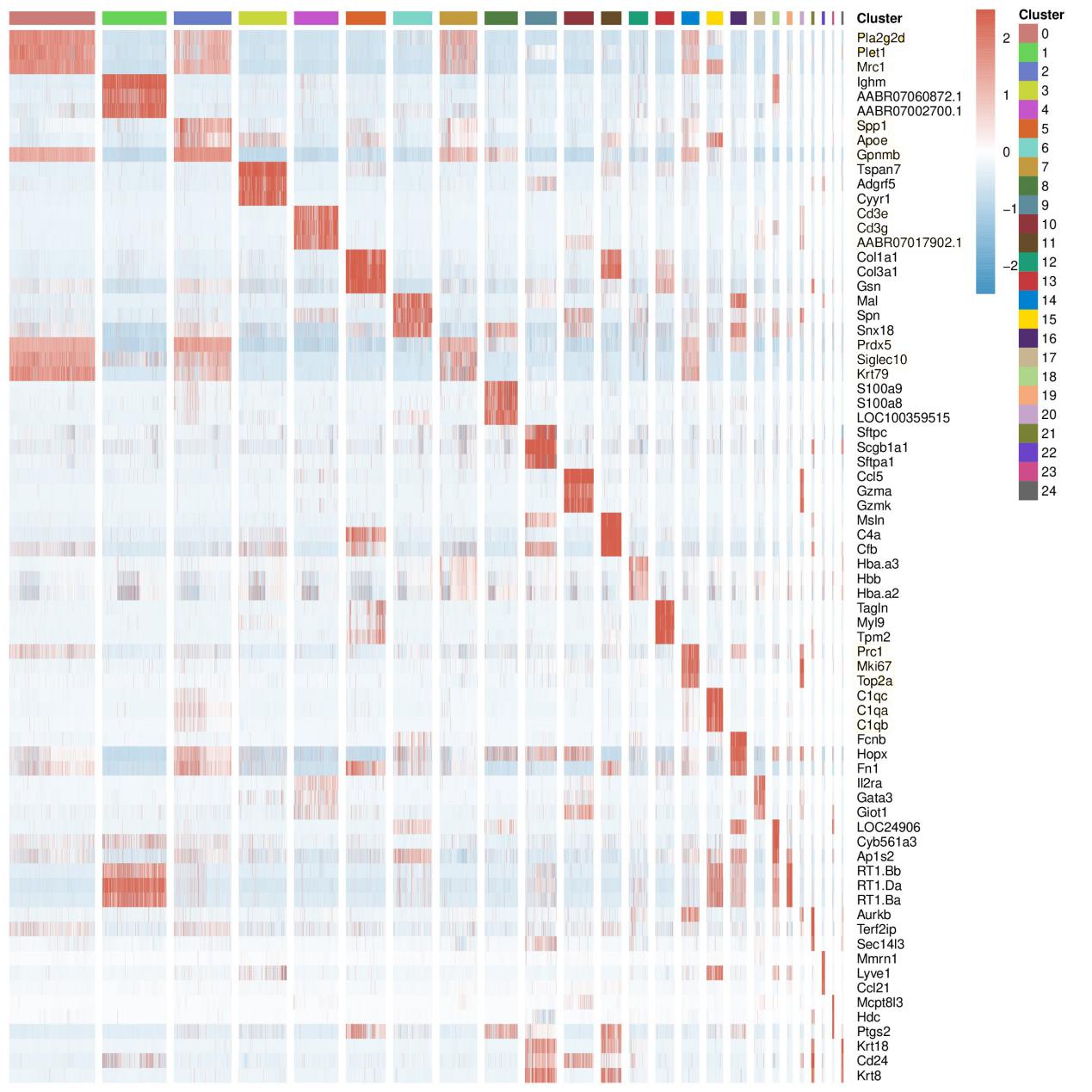


**Supplementary Figure 3. Marker genes of recognized cell clusters.**

Heatmap of the top 3 differentially expressed genes within 25 recognized cell clusters.


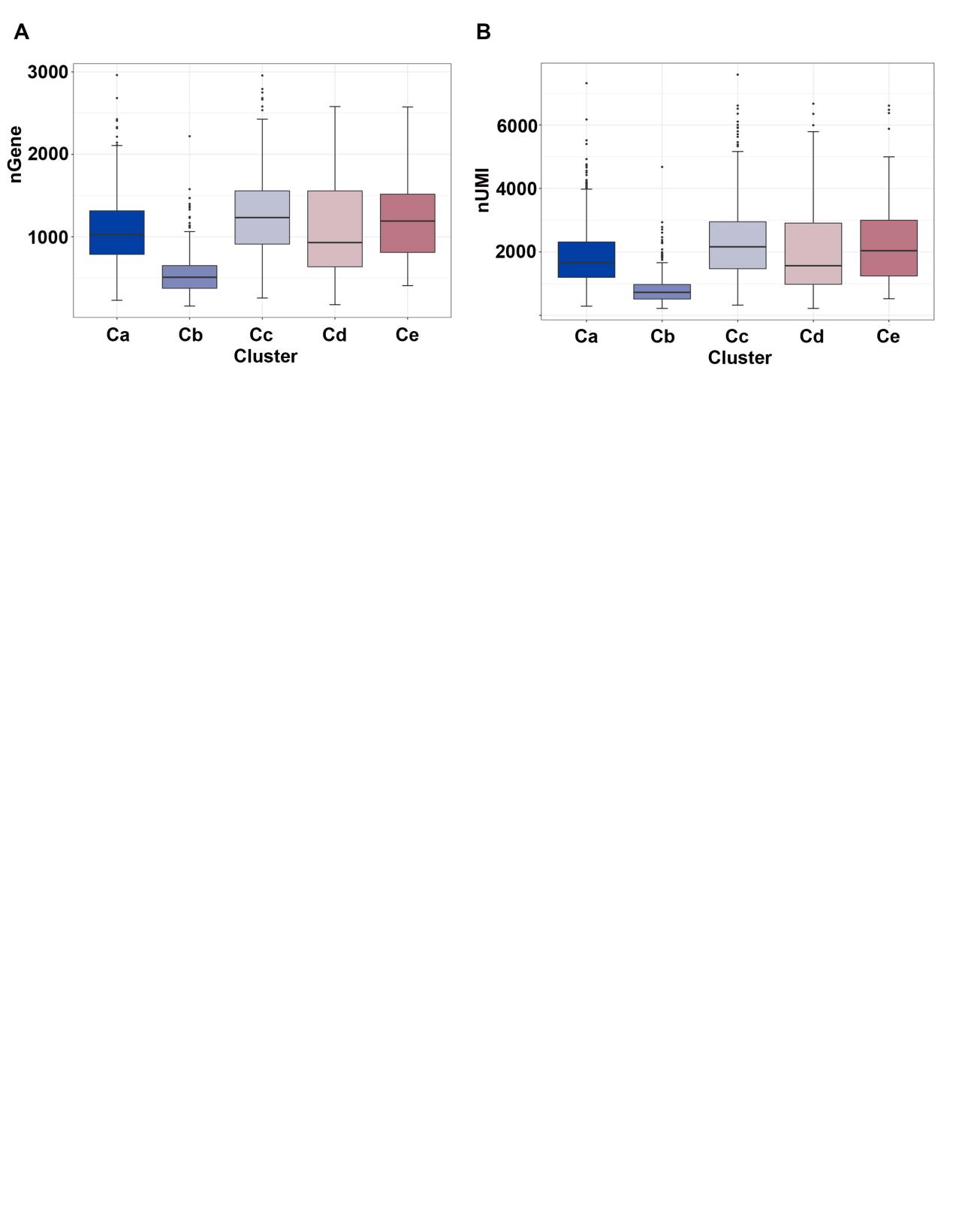


**Supplementary Figure 4. Transcript abundance and gene number of EC clusters.**

(A) Statistics of the number of genes in EC clusters. (B) Statistics of the transcript abundance of EC clusters.


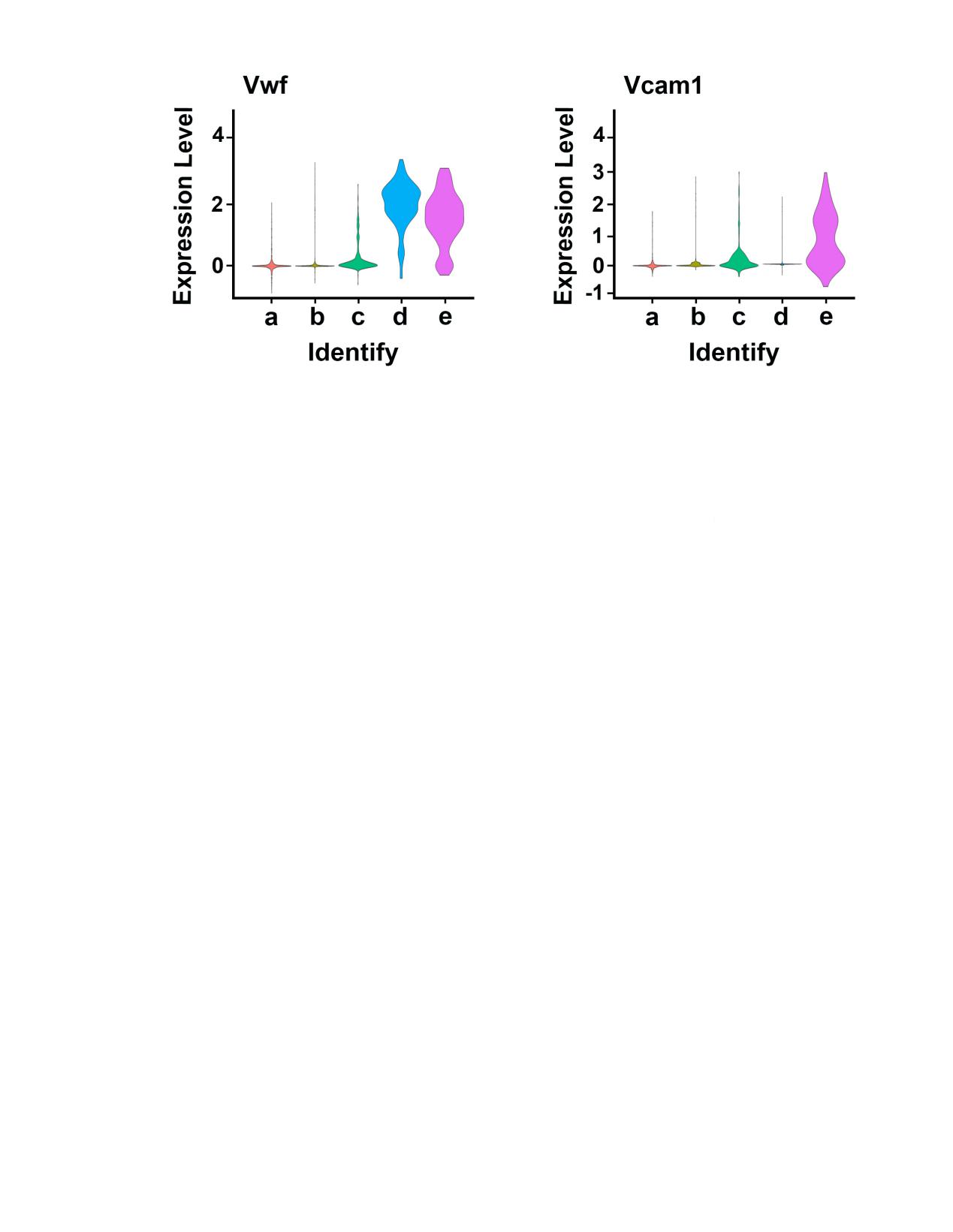


**Supplementary Figure 5. The expression level of Vcam1 in EC clusters.**

Violin plots of the expression level of Vcam1 (Vascular cell adhesion protein 1) in five EC clusters.


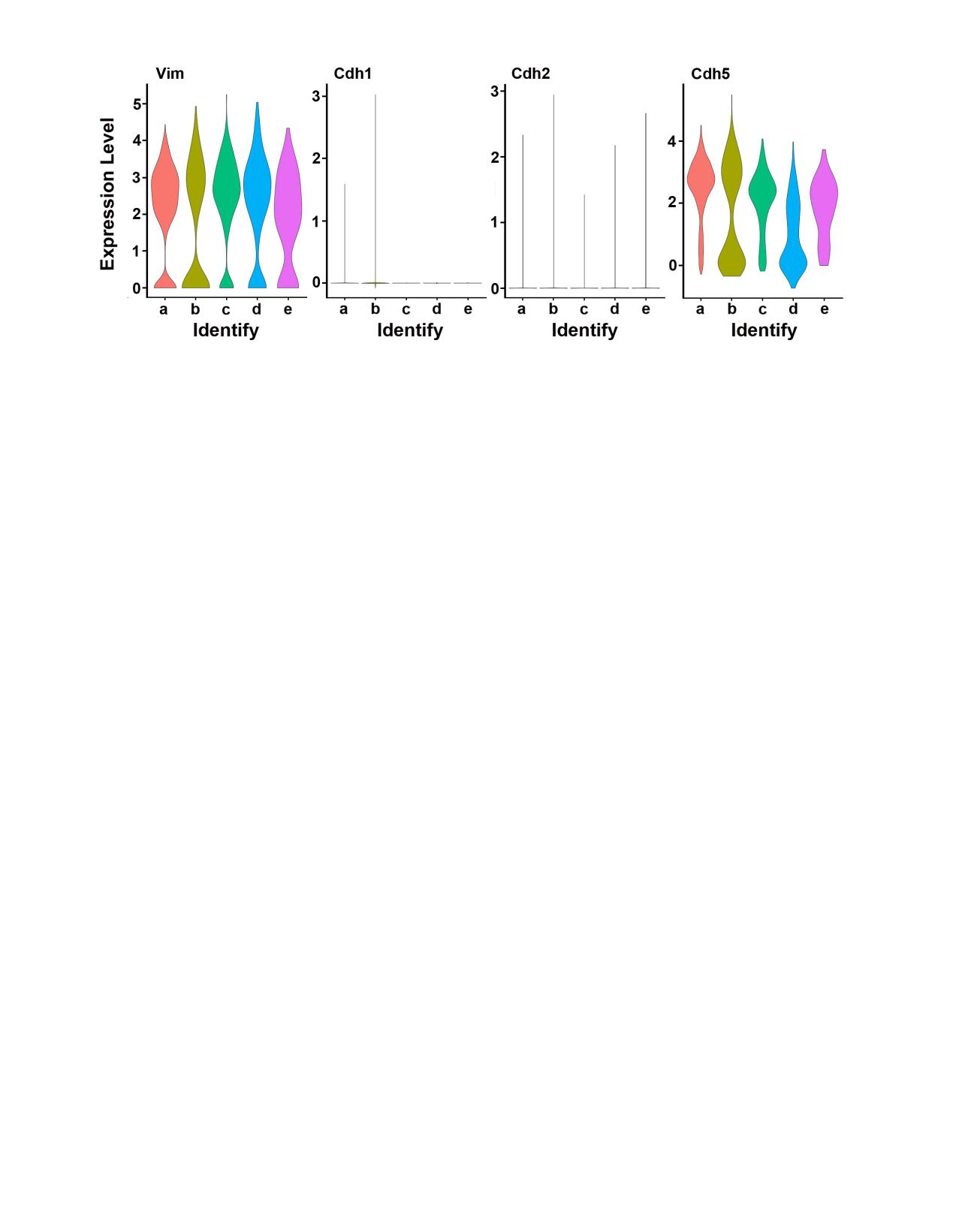


**Supplementary Figure 6. The expression levels of EndMT related genes in EC clusters.**

Violin plots of the expression levels of Vim (encoding Vimentin), Cdh1 (Cadherin-1/E-cadherin), Cdh2 (Cadherin-2/N-cadherin) and Cdh5 (Cadherin-5/VE-cadherin).


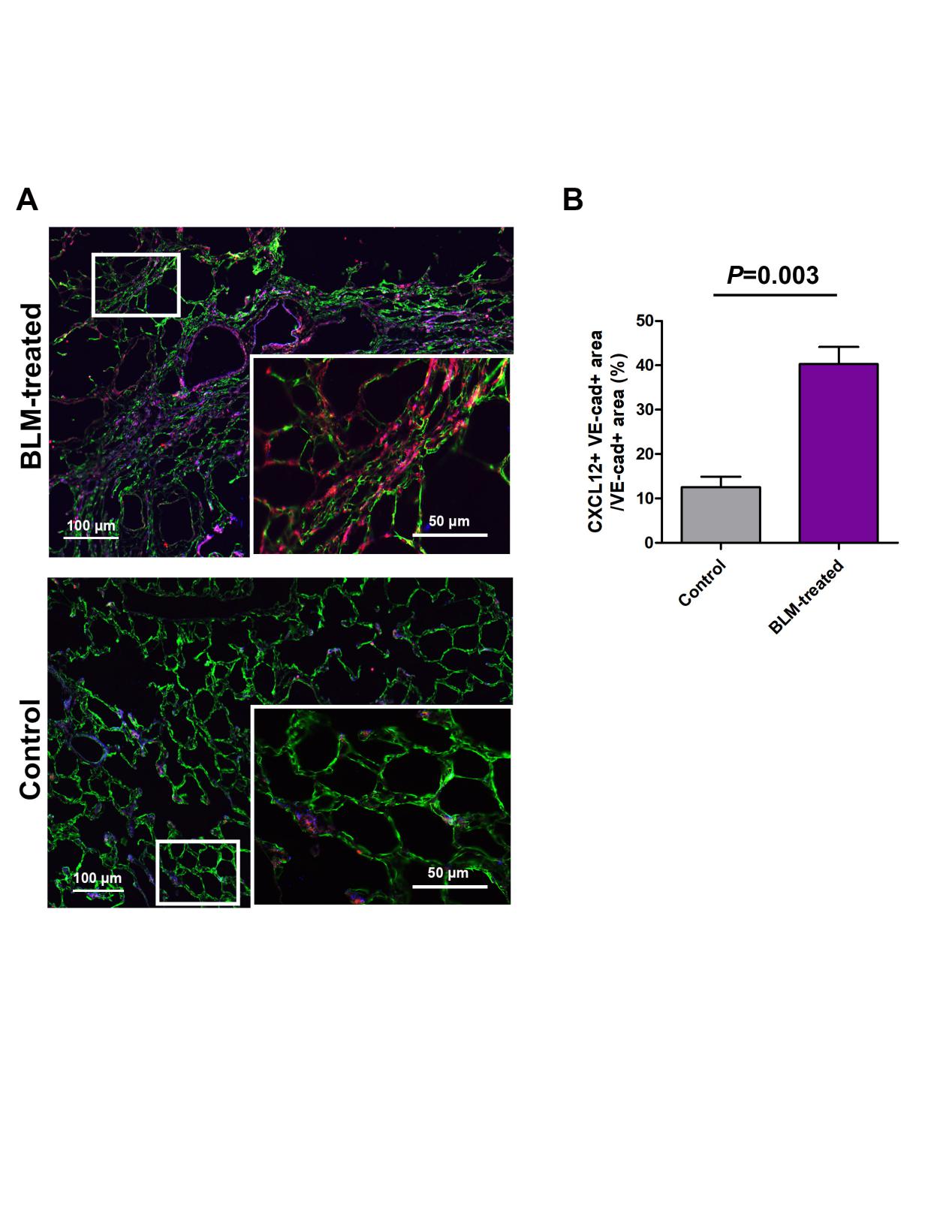


**Supplementary Figure 7. Localization of cluster *a* ECs in BLM-treated and control lungs**

(A) Representative immunofluorescence image: Cxcl12 (red), VE-cadherin (green), DAPI (blue), bar (left)=100 μm, bar (right)=50 μm. (B) Quantitative analysis of CXCL12+ ECs. Data is shown as Mean ± SEM. Statistical analysis using Mann-Whitney two-tailed test (n=5).


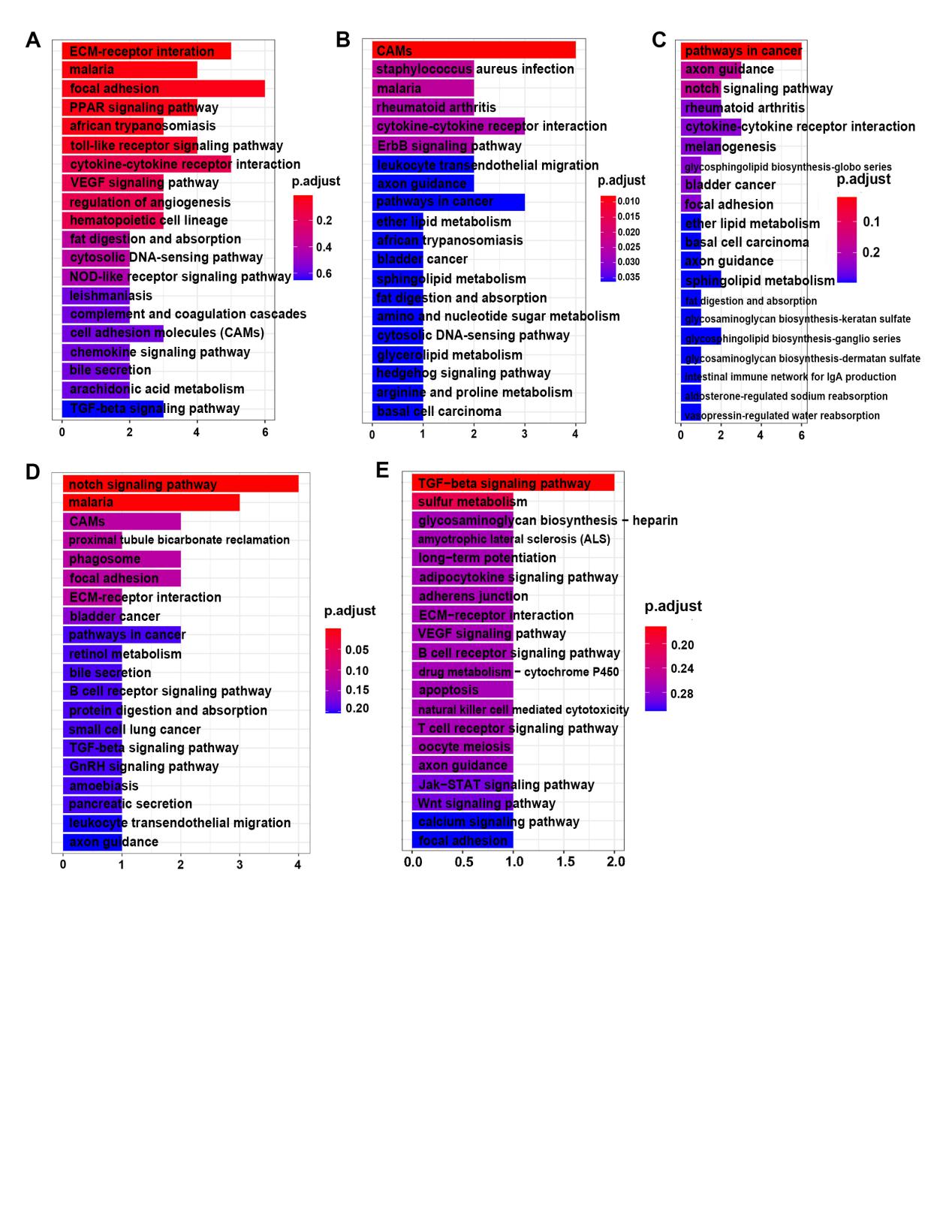


**Supplementary Figure 8. KEGG analysis on genes that were highly expressed in EC clusters.**

(A-E) Pathway enrichment analysis (top 20) of cluster *a, b, c, d* and *e* ECs, Fisher’s exact test.


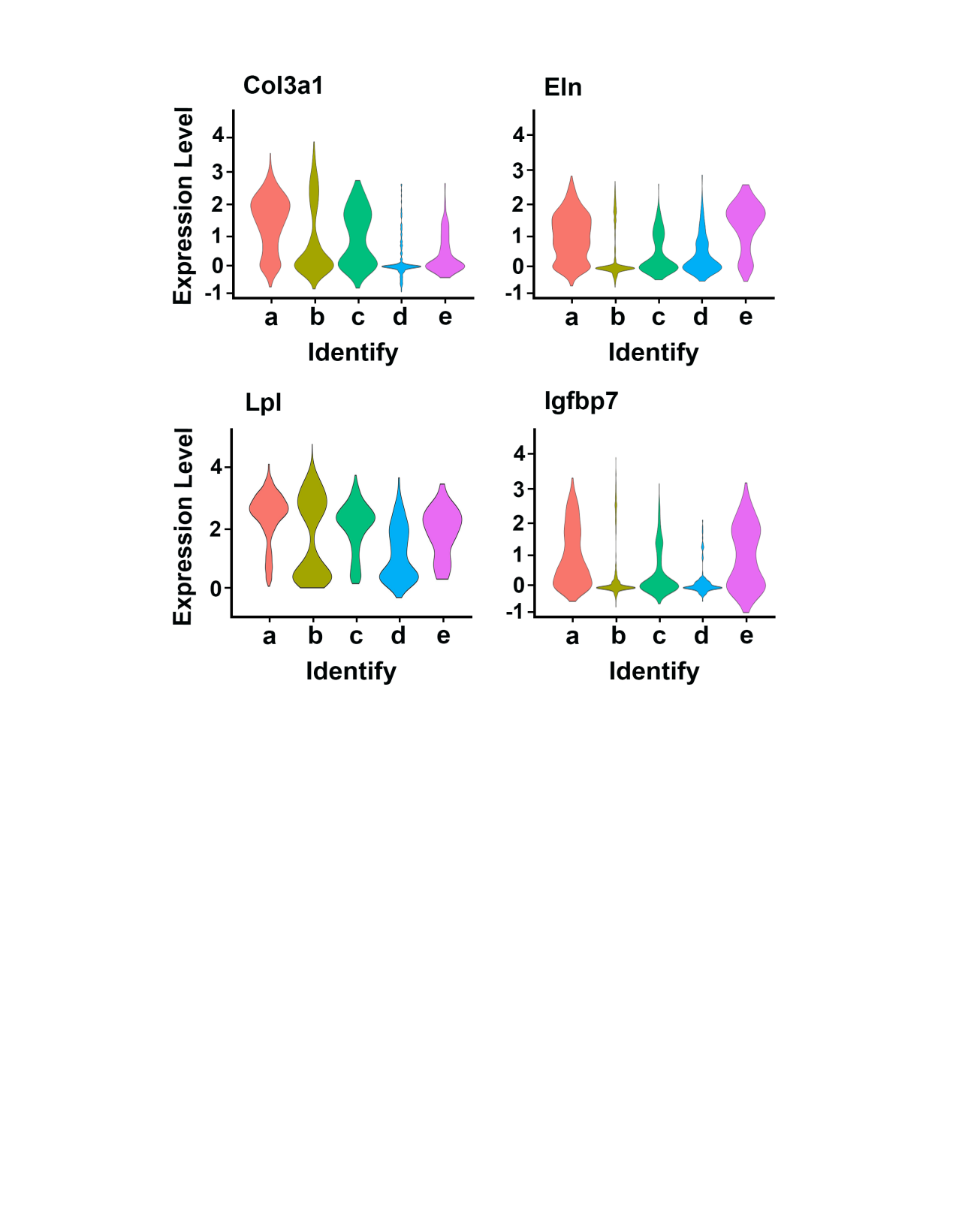


**Supplementary Figure 9. The expression levels of ECM-related genes in cluster *a* ECs.**

Violin plots of the expression levels of Col3a1 (Collagen alpha-1 (III) chain), Eln (Elastin), Lpl (Lipoprotein lipase) and Igfbp7 (Insulin-like growth factor-binding protein 7).


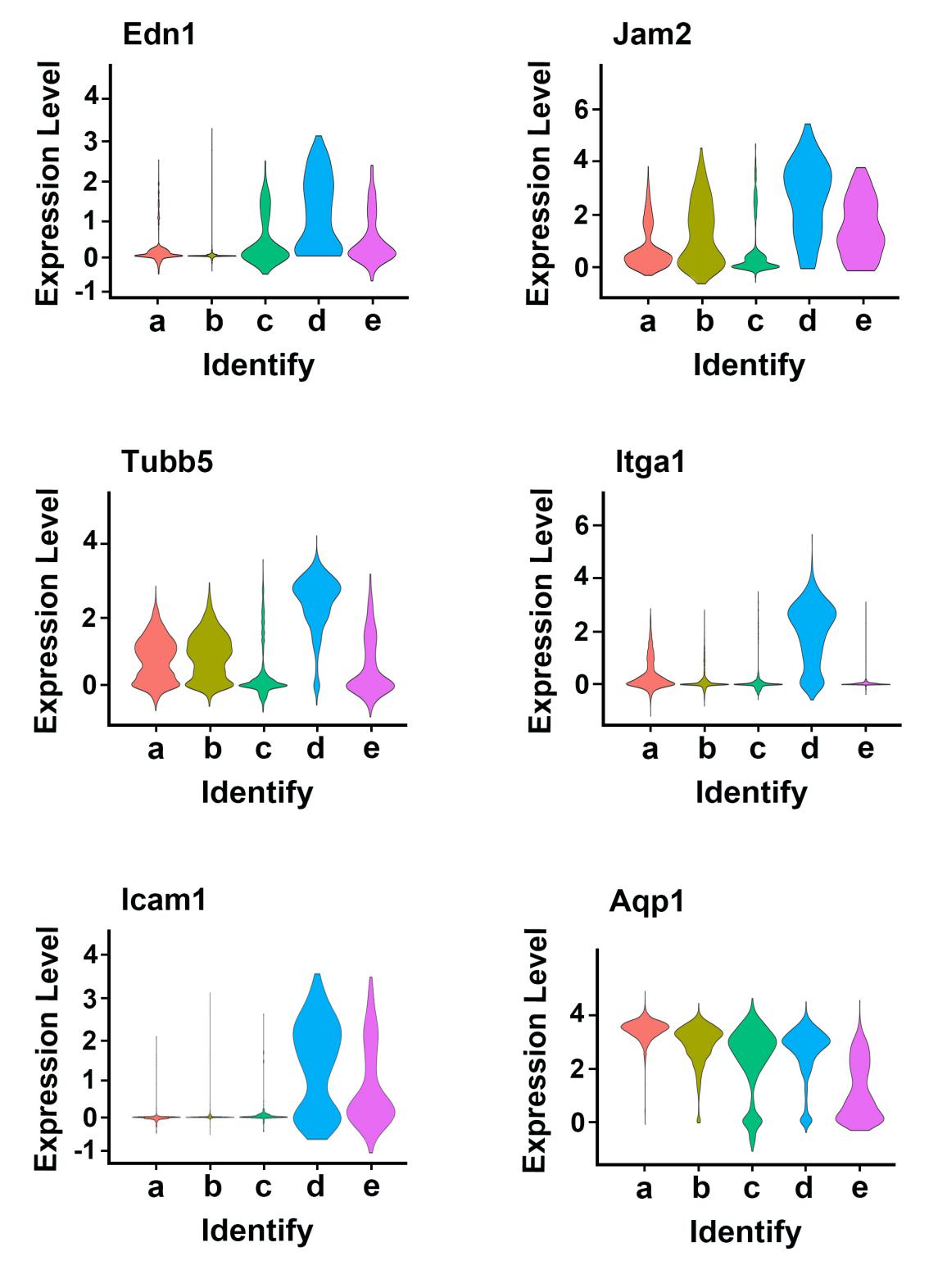


**Supplementary Figure 10. Genes related to process of transmitting mechanical stress into biochemical signaling in cluster *d* ECs.**

Violin plots of the expression levels of Edn1 (encoding endothelin-1), Jam2 (Junctional adhesion molecule B), Tubb5 (Tubulin beta-5 chain), Itga1 (Integrin alpha-1), Icam1 (Intercellular adhesion molecule 1) and Aqp1 (Aquaporin-1).


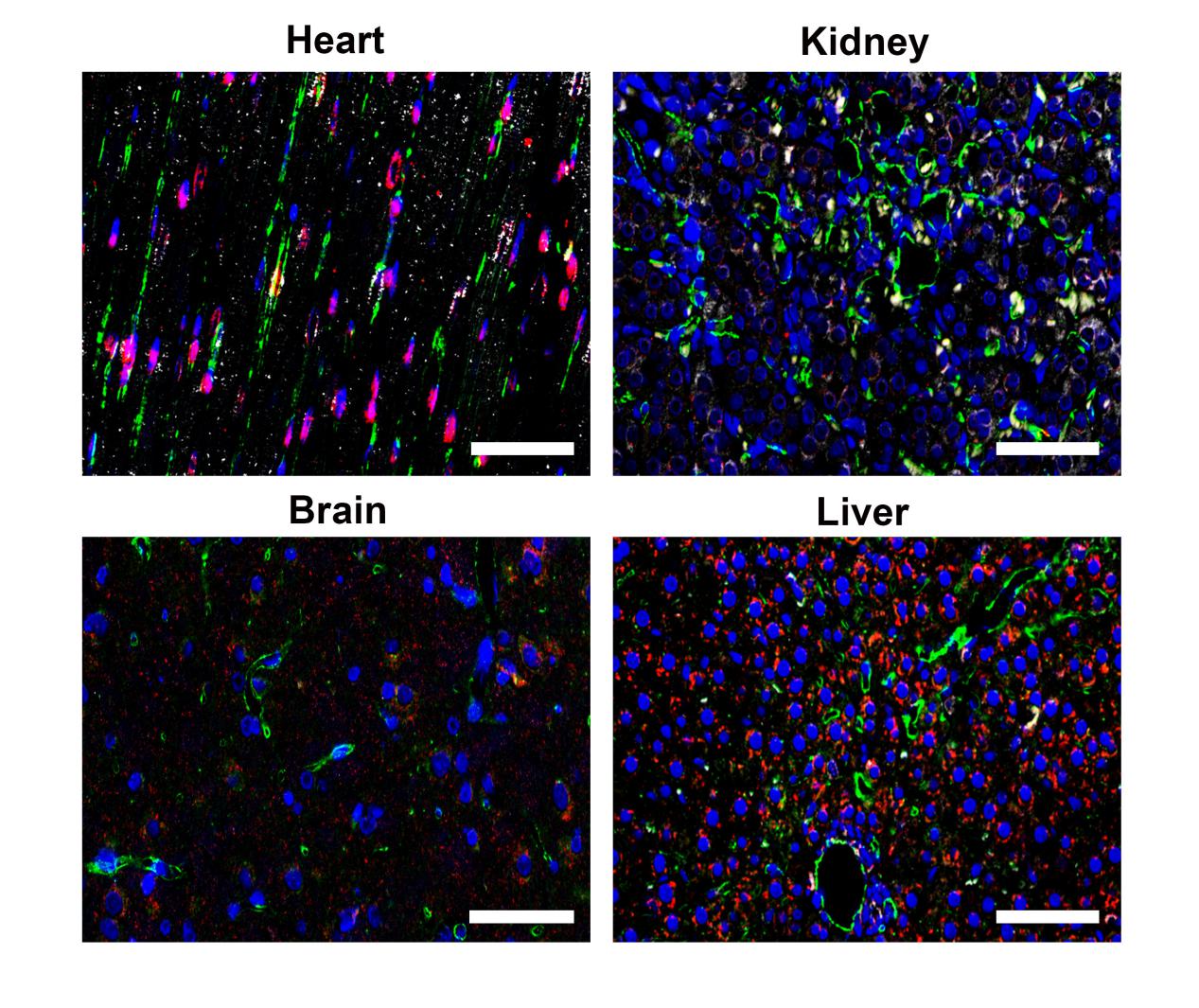


**Supplementary Figure 11. Identification of cluster *a* ECs in rat heart, liver, brain and kidney.**

Representative immunofluorescence image: Nos (red), Cav1 (white), VE-cadherin (green), DAPI (blue), bar=50 μm.


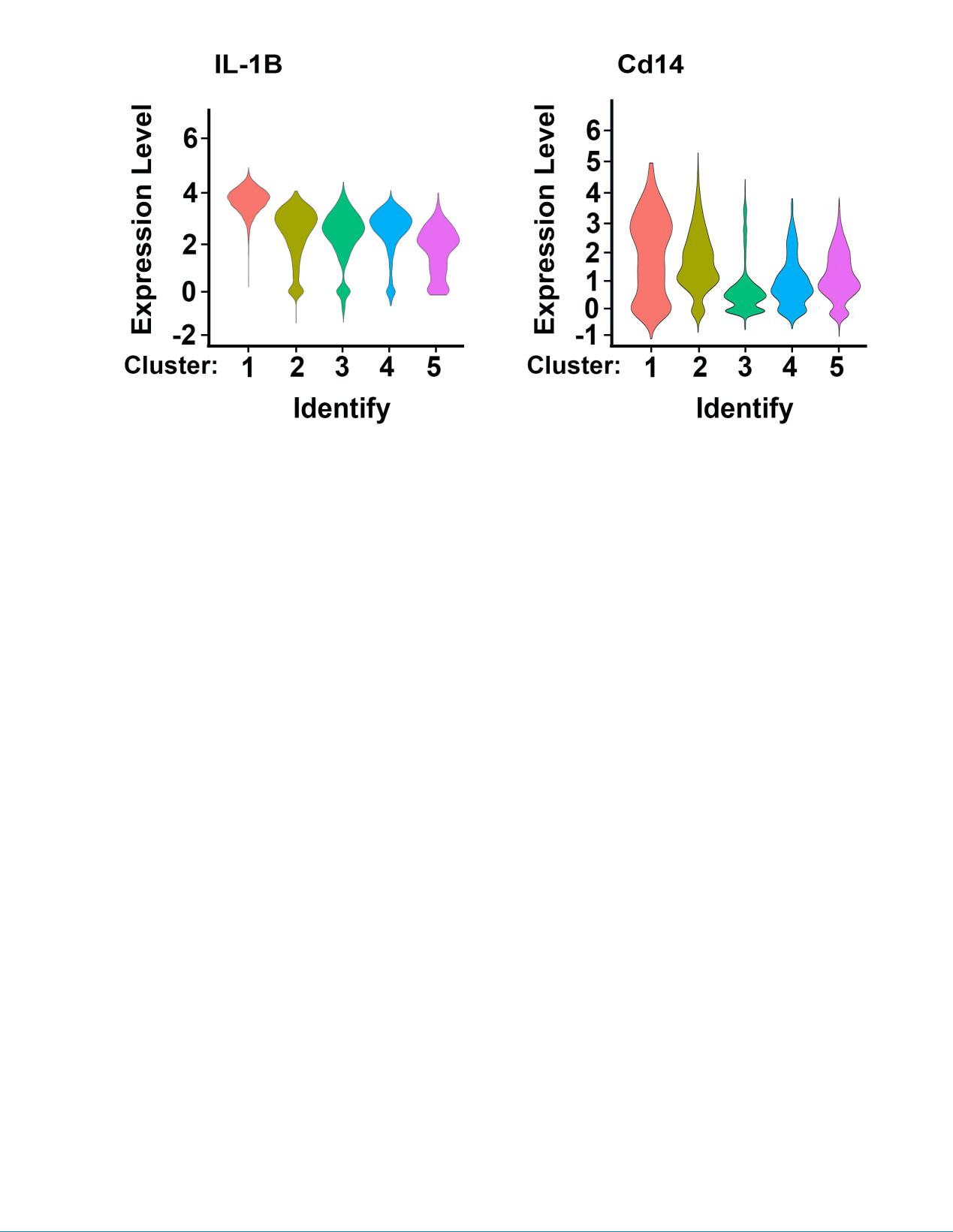


**Supplementary Figure 12. The expression levels of marker genes associated with monocytes.**

Violin plots of the expression levels of IL-1B (encoding interleukin-1 beta) and Cd14 (encoding monocyte differentiation antigen CD14).


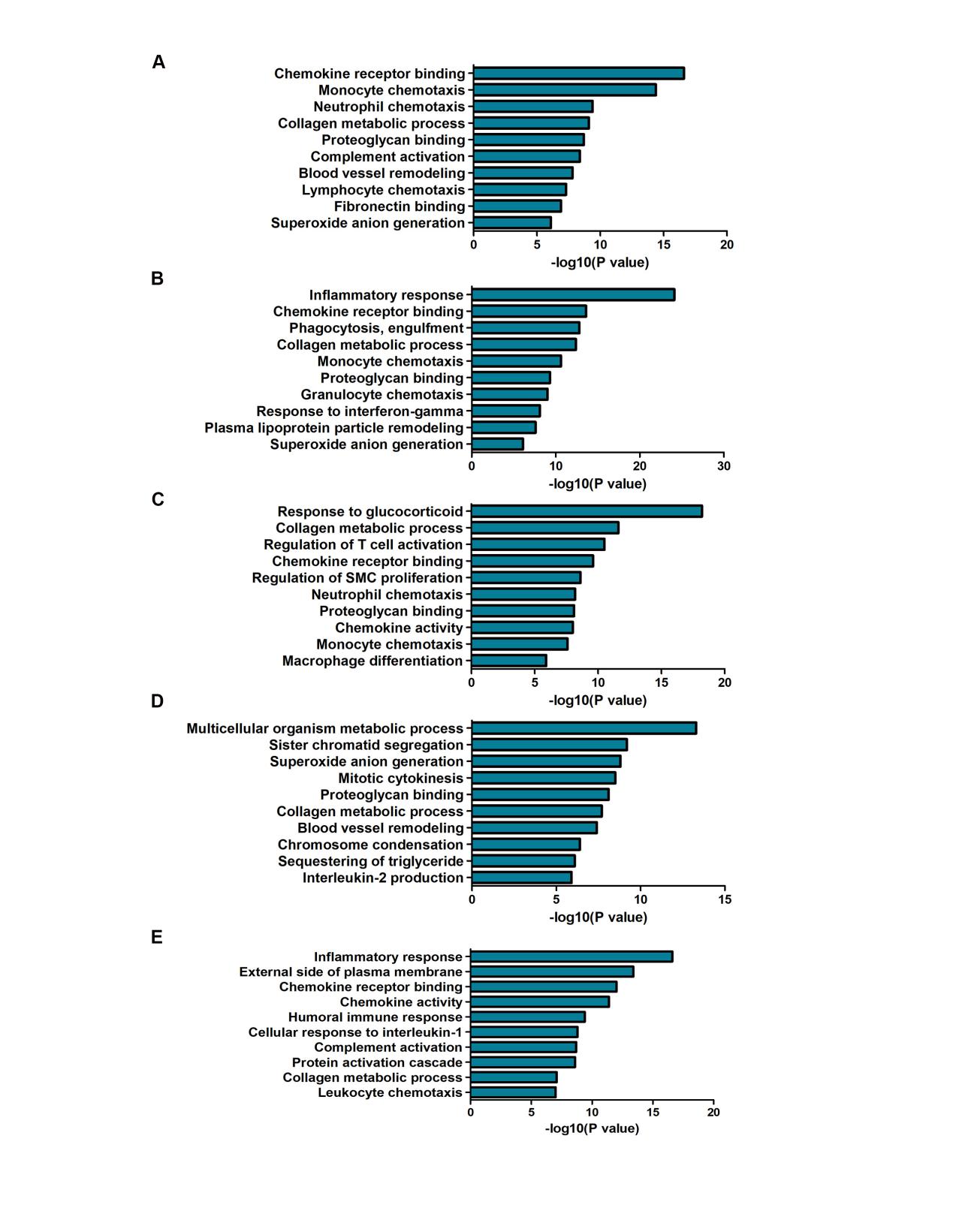


**Supplementary Figure 13. Gene Ontology enrichment analysis on genes that are highly expressed in macrophage clusters.**

(A) Selected GO enrichment of cluster *1* macrophages. (B) Selected GO enrichment of cluster *2* macrophages. (C) Selected GO enrichment of cluster *3* macrophages. (D) Selected GO enrichment of cluster *4* macrophages. (E) Selected GO enrichment of cluster *5* macrophages. Fisher’s exact test.


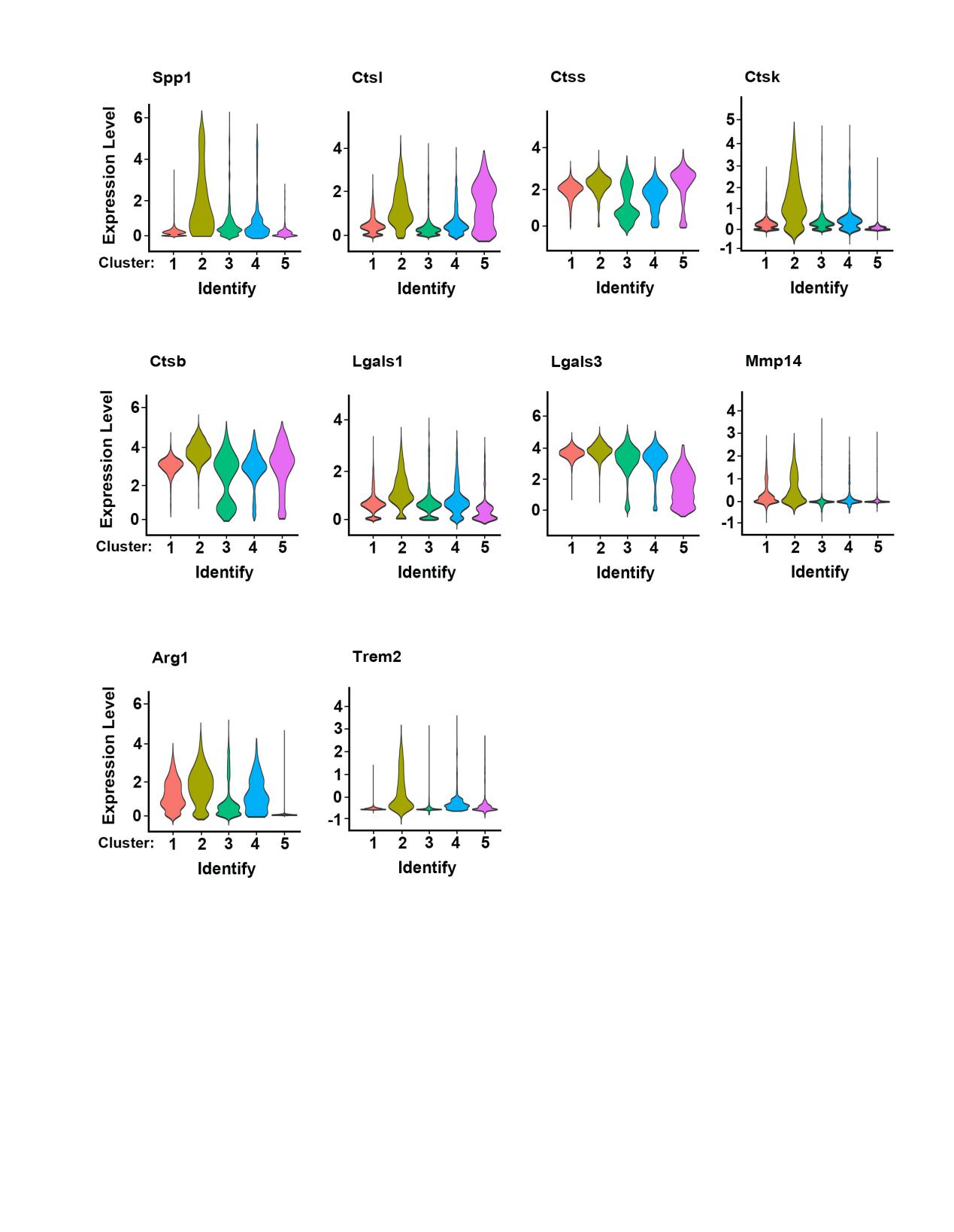


**Supplementary Figure 14. The expression levels of scar-associated genes in cluster *2* macrophages.**

Violin plots of the expression levels of Spp1 (encoding osteopontin), Ctsl (procathepsin L), Ctss (cathepsin S), Ctsb (cathepsin b), Lgals1 (galectin-1), Lgals3 (galectin-3), Mmp14 (matrix metalloproteinase-14), Arg1 (arginase-1) and Trem2 (triggering receptor expressed on myeloid cells 2).


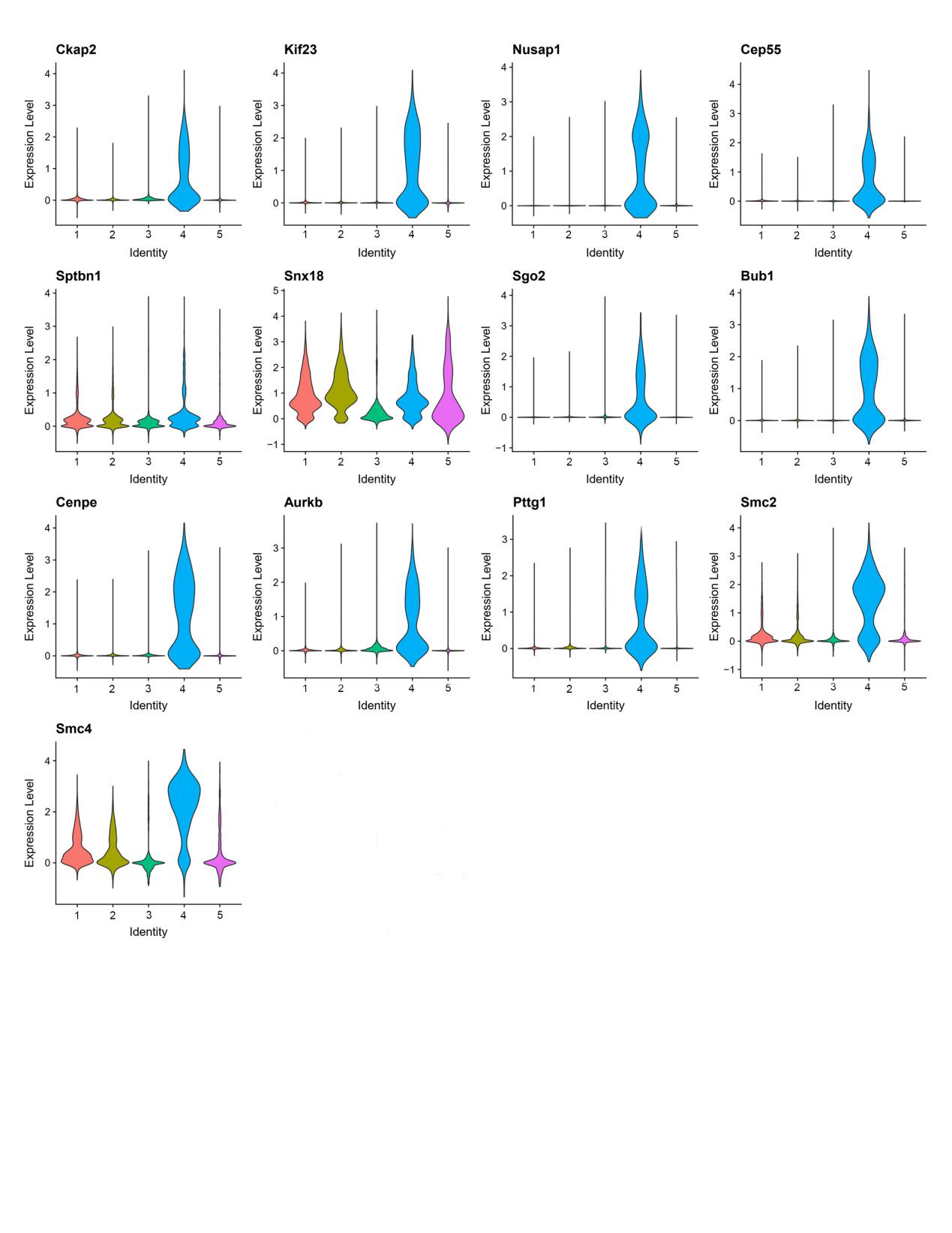


**Supplementary Figure 15. The expression levels of selected genes in cluster *4* macrophages.**

Violin plots of the expression levels of Ckap2 (encoding cytoskeleton-associated protein 2), Kif23 (kinesin-like protein)，Nusap1 (nucleolar and spindle-associated protein 1), Cep55 (centrosomal protein of 55 kDa), Sptbn1 (spectrin beta chain), Snx18 (sorting nexin-18), Sgo2 (shugoshin 2), Bub1 (mitotic checkpoint serine/threonine-protein kinase BUB1), Cenpe (centromere-associated protein E), Aurkb (aurora kinase b), Pttg1 (pituitary tumor-transforming gene 1), and Smc2 (SMC protein 2).


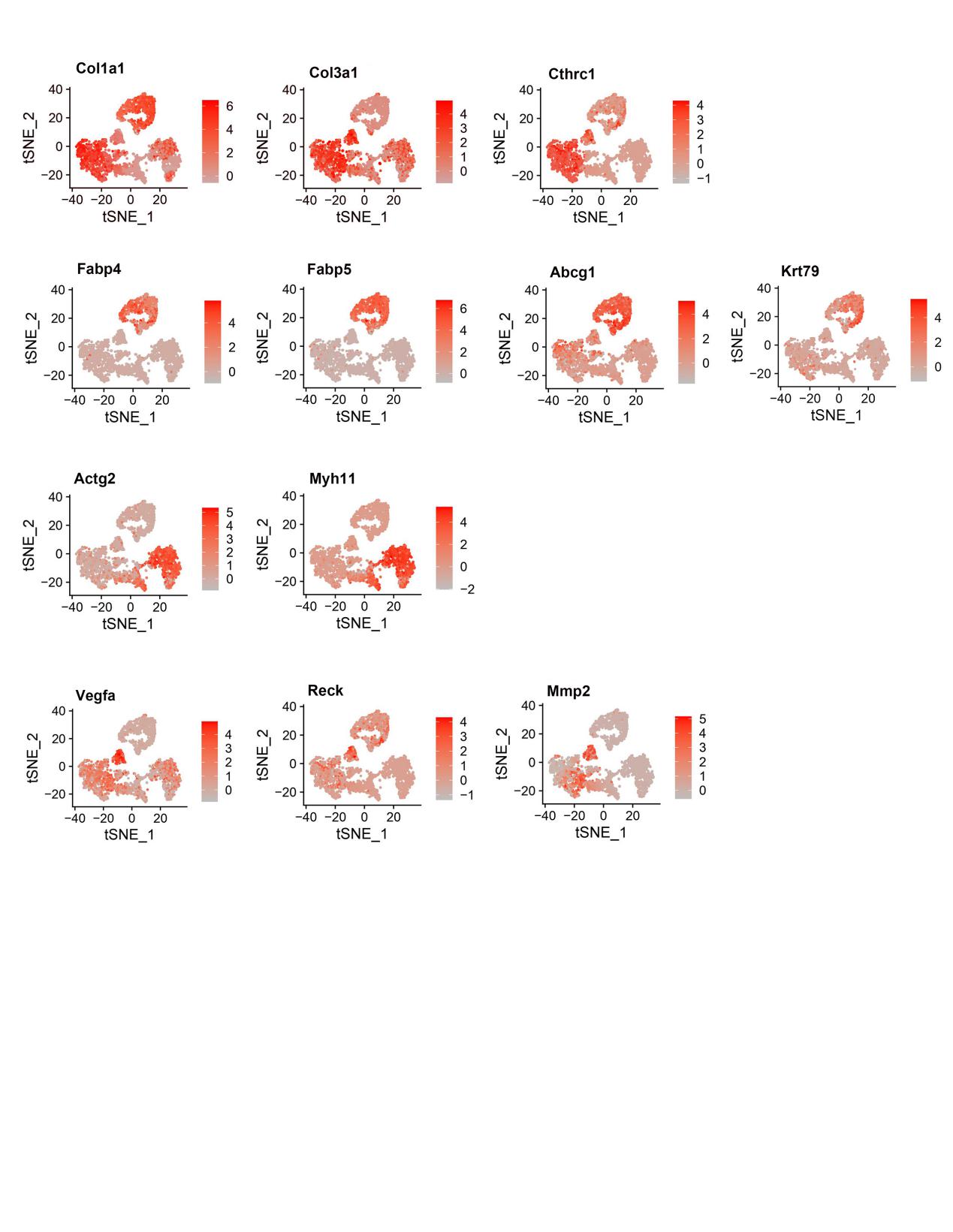


**Supplementary Figure 16. The expression levels of selected genes in stromal cell clusters.**

Expression levels of Col1a1 (encoding collagen alpha-1 (I) chain), Col3a1 (collagen alpha-1 (III) chain), Cthrc1 (collagen triple helix repeat-containing protein 1), Fabp4 (fatty acid-binding protein-4), Fabp5 (fatty acid-binding protein-5), Abcg1 (ATP-binding cassette sub-family G member 1), Krt79 (type II cytoskeletal 79), Actg2 (actin gamma 2), Myh11 (myosin-11), Vegfa (vascular endothelial growth factor A), Reck (reversion-inducing cysteine-rich protein with Kazal motifs), Mmp2 (type IV collagenase) on t-SNE plots.


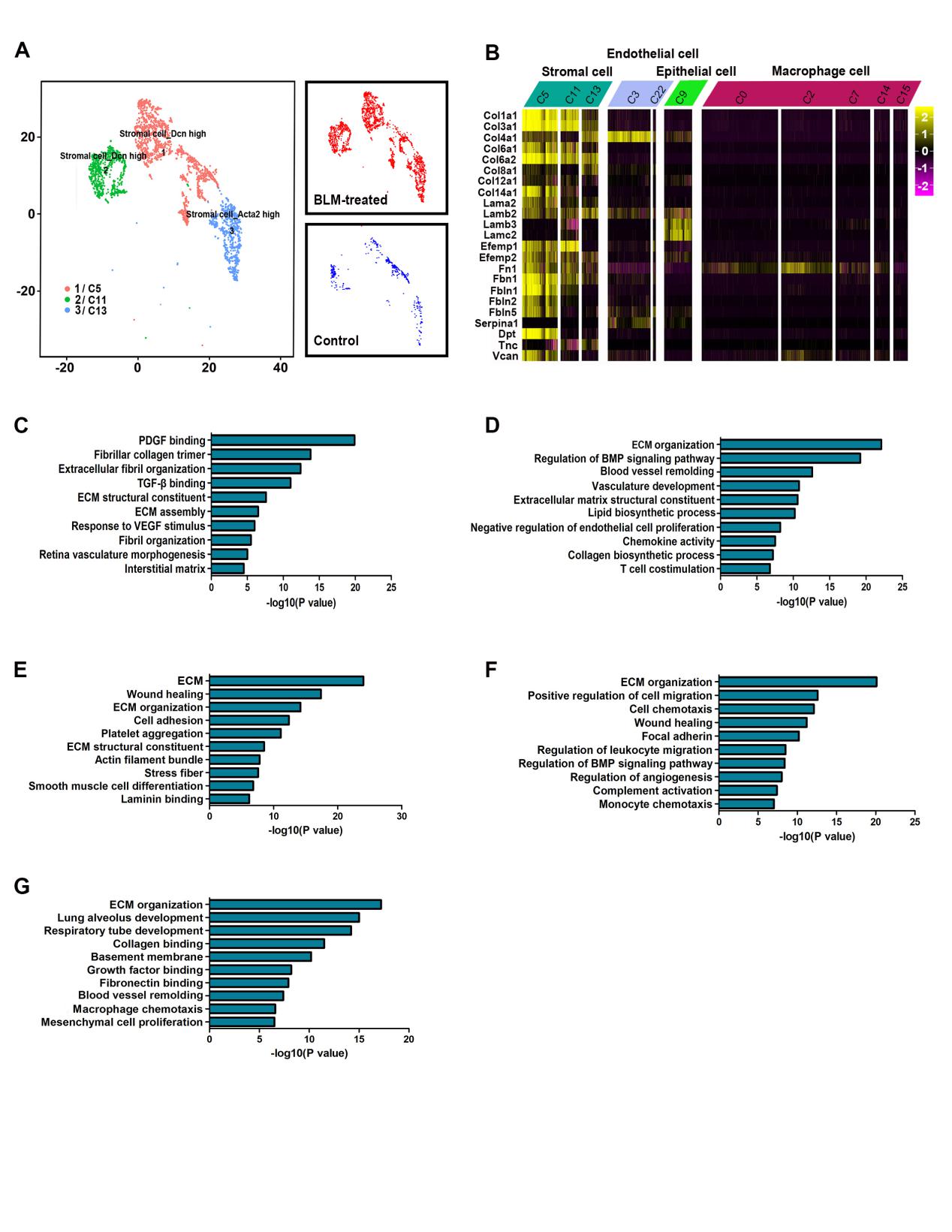


**Supplementary Figure 17. Single-cell profiling of stromal cells and GO analysis.**

(A) Clustering and annotating 3,067 stromal cells (left), annotating injury condition (right). (B) Heatmap depicting relative expression (normalized and scaled z-scored) of known ECM components previously shown to be increased in fibrotic lungs. (C) Selected GO enrichment of cluster *1* stromal cells. (D) Selected GO enrichment of cluster *2* stromal cells. (E) Selected GO enrichment of cluster *3* stromal cells. (F) Selected GO enrichment of cluster *4* stromal cells. (G) Selected GO enrichment of cluster *5* stromal cells. Fisher’s exact test.


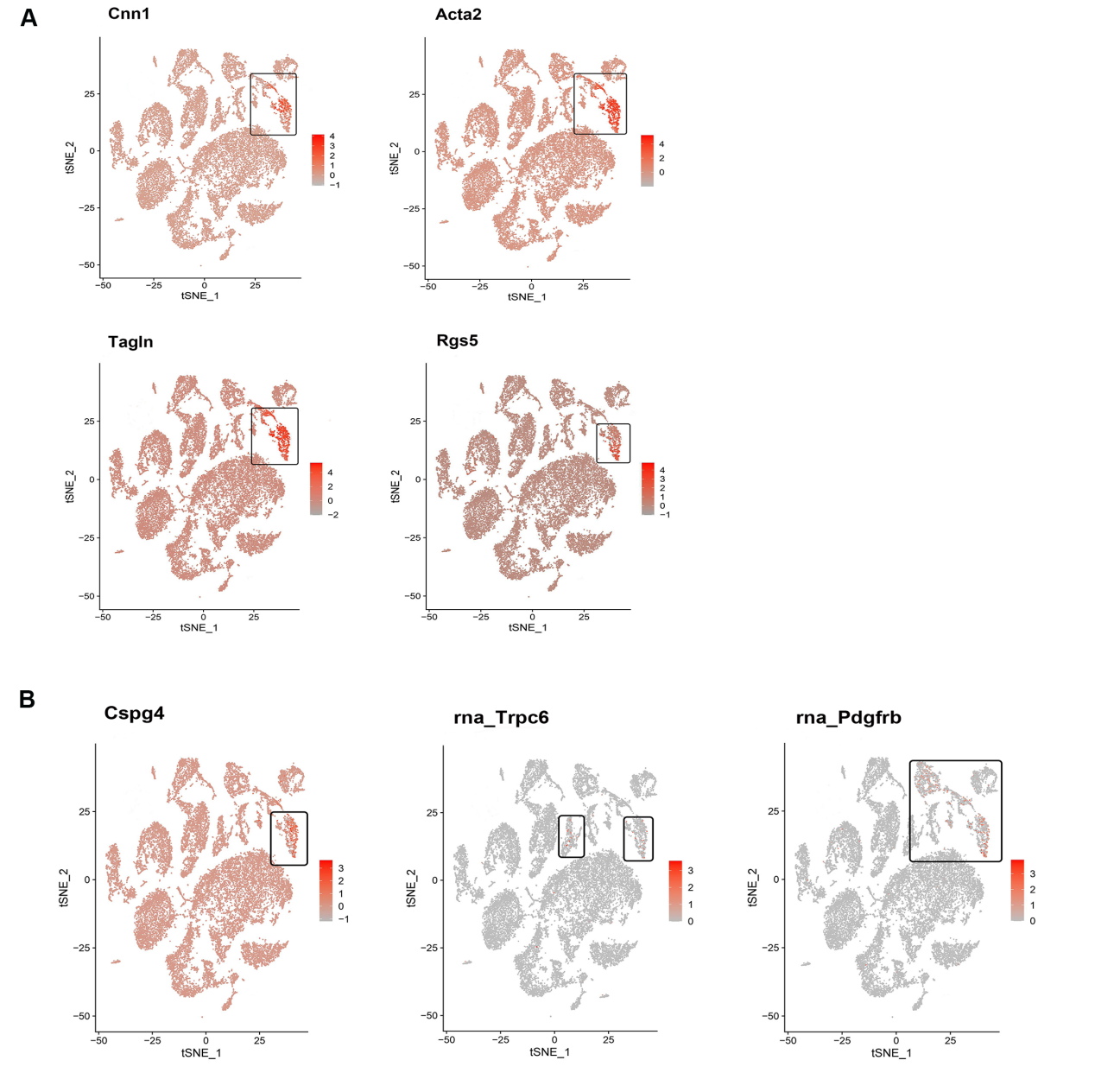


**Supplementary Figure 18. The expression of selected genes in 25,547 cells from the control and BLM-treated lungs.**

1. Distribution of known marker genes of vascular smooth muscle cells. (B) Distribution of marker genes of perivascular cells.
